# Sex Differences in Colon Cancer Metabolism Reveal A Novel Subphenotype

**DOI:** 10.1038/s41598-020-61851-0

**Published:** 2020-03-17

**Authors:** Yuping Cai, Nicholas J. W. Rattray, Qian Zhang, Varvara Mironova, Alvaro Santos-Neto, Kuo-Shun Hsu, Zahra Rattray, Justin R. Cross, Yawei Zhang, Philip B. Paty, Sajid A. Khan, Caroline H. Johnson

**Affiliations:** 1 Department of Environmental Health Sciences, Yale School of Public Health, Yale University, New Haven, CT USA; 2 Strathclyde Institute of Pharmacy and Biomedical Sciences, University of Strathclyde, Glasgow, UK; 3 Department of Surgery, Memorial Sloan Kettering Cancer Center, New York, NY USA; 4 Cancer Biology and Genetics Program, Memorial Sloan-Kettering Cancer Center, New York, NY USA; 5 Department of Surgery, Yale University School of Medicine, New Haven, CT USA; 6 Department of Surgery, Division of Surgical Oncology, Yale University School of Medicine, New Haven, CT USA

**Keywords:** Colon cancer, Cancer metabolism

## Abstract

Women have a lower incidence of colorectal cancer (CRC) than men, however, they have a higher incidence of right-sided colon cancer (RCC). This is of concern as patients with RCC have the poorest clinical outcomes among all CRC patients. Aberrant metabolism is a known hallmark and therapeutic target for cancer. We propose that metabolic subphenotypes exist between CRCs due to intertumoral molecular and genomic variation, and differences in environmental milieu of the colon which vary between the sexes. Metabolomics analysis of patient colon tumors (n = 197) and normal tissues (n = 39) revealed sex-specific metabolic subphenotypes dependent on anatomic location. Tumors from women with RCC were nutrient-deplete, showing enhanced energy production to fuel asparagine synthesis and amino acid uptake. The clinical importance of our findings were further investigated in an independent data set from The Cancer Genomic Atlas, and demonstrated that high asparagine synthetase (*ASNS*) expression correlated with poorer survival for women. This is the first study to show a unique, nutrient-deplete metabolic subphenotype in women with RCC, with implications for tumor progression and outcomes in CRC patients.

## Introduction

Colorectal cancer (CRC) is the third most commonly diagnosed cancer in the United States, and the second leading cause of cancer-related deaths for cancers that affect both men and women^1^. In 2019, there will be an estimated 145,600 new cases and 51,020 deaths attributed to the disease^2^. Cancers of the colorectum can be categorized by anatomic location. Right-sided colon cancers (RCCs) occur in the cecum, ascending colon and hepatic flexure, and left-sided colon cancers (LCCs) occur in the splenic flexure, descending, sigmoid and rectosigmoid colon. Mid and lower rectal cancers are sometimes grouped as LCCs^3^. Tumors in different anatomic locations have differing clinical outcomes, and recent epidemiologic studies have shown that patients with RCC have the poorest survival, even when adjusted for confounding factors, including clinical stage^4–6^. This suggests that differences exist in the underlying tumor biology based on anatomic location. Distinguishing molecular features of RCCs include high microsatellite instability (MSI-H), valine to glutamate *BRAF* mutations at codon 600, cytosine-guanosine (CpG) island methylation phenotype (CIMP)^7,8^, and diploid cells^9^. In contradistinction, patients with LCCs more frequently have chromosomal instability, mutations in the *TP53* and *APC* genes, and aneuploidy^10^. Recently, expression arrays have revealed four molecular subtypes of CRCs, called “consensus molecular subtypes” which tend to occur more frequently in either the right- or left-sides of the colon. Consensus molecular subtype 1, for example, has a 77% frequency in RCCs and is enriched for high MSI, CIMP, and *BRAF* mutations. In addition, gene set enrichment analysis shows molecular pathways related to immune infiltration and PD-1 activation for this subtype^10,11^.

In addition to genetic variance in colorectal subsites, RCCs occur more frequently in women compared to men, whereas LCCs have an equal frequency between sexes. Data from the Clinical Outcomes Research Initiative (CORI) and Surveillance, Epidemiology, and End Results (SEER) databases show that the percentage of RCC cases is strikingly higher in women than in men (61.7% vs 38.3%), while only slightly more LCC cases are observed in women than men (52.1% vs 47.8%)^9^. The reason for this sex difference is unclear and of concern for women, due to the association of RCCs with poorer clinical outcome.

Recent studies have indicated that in addition to genetic factors, the environmental milieu could differ along the length of the colorectum and influence tumor development and progression^10^. This milieu is comprised of metabolites produced from the diet, microbiome, and environmental pollutants^7,12–14^. Sex-specific influences on metabolite production could additionally account for the differences observed in anatomical tumor location. Life-course exposure to sex-steroid hormones (estrogens and androgens), lifestyle and medication use (diet, physical activity, pregnancy, oral contraceptives) and microbiome diversity and metabolism, could contribute to specific metabolic phenotypes of colon cancer between men and women^15–18^. Compared to men, women have a much lower overall incidence of CRC, yet they are more frequently diagnosed with RCC, thus it is highly plausible that RCCs have a unique biology in women shaped by differences in life-course exposures which promotes their growth in this location and affects therapeutic response.

Aberrant metabolism, which is a salient feature of colon tumor cells, involves the alteration of metabolic pathways to increase macromolecules and energy for cell growth. Metabolic pathways known to be affected in tumor cells include glycolysis, glutaminolysis, one-carbon metabolism, and fatty acid synthesis^19,20^. With advancements in high-throughput analytical techniques for metabolomics research, there are emerging studies that have revealed metabolic signatures in colon cancer^21,22^. However, there have been no studies investigating sex-associated differences in colon cancer metabolism. More importantly, it is not known if men and women have metabolic phenotypes specific to the anatomic location of the colon tumor.

In this study, comprehensive untargeted metabolomics analysis was performed on normal colon and primary colon tumor tissues collected from a large cohort of CRC patients. Multiple comparative analyses were conducted identifying sex differences in colon tumor metabolites stratified by anatomic location and stage. The biological relevance of these sex-specific colon cancer metabolites was identified by understanding their involvement in metabolic pathways. We identified sex-specific differences in energy production, and widespread sex and anatomic differences in asparagine, methionine, and polyamine metabolism. We correlated our findings to clinical outcomes of patients with colon cancer using The Cancer Genome Atlas (TCGA) database, and identified a positive association of high asparagine synthesis and poorer survival in women with colon cancer. To our knowledge, this is the first high-throughput metabolomic study to identify sex differences in colon cancer metabolism. We have revealed novel insights into the biological differences in tumor metabolism within population groups, and identified asparagine metabolism as potential future therapeutic target for women with RCC.

## Results

### Sex-specific differences in tumor metabolites

**Figure 1:**
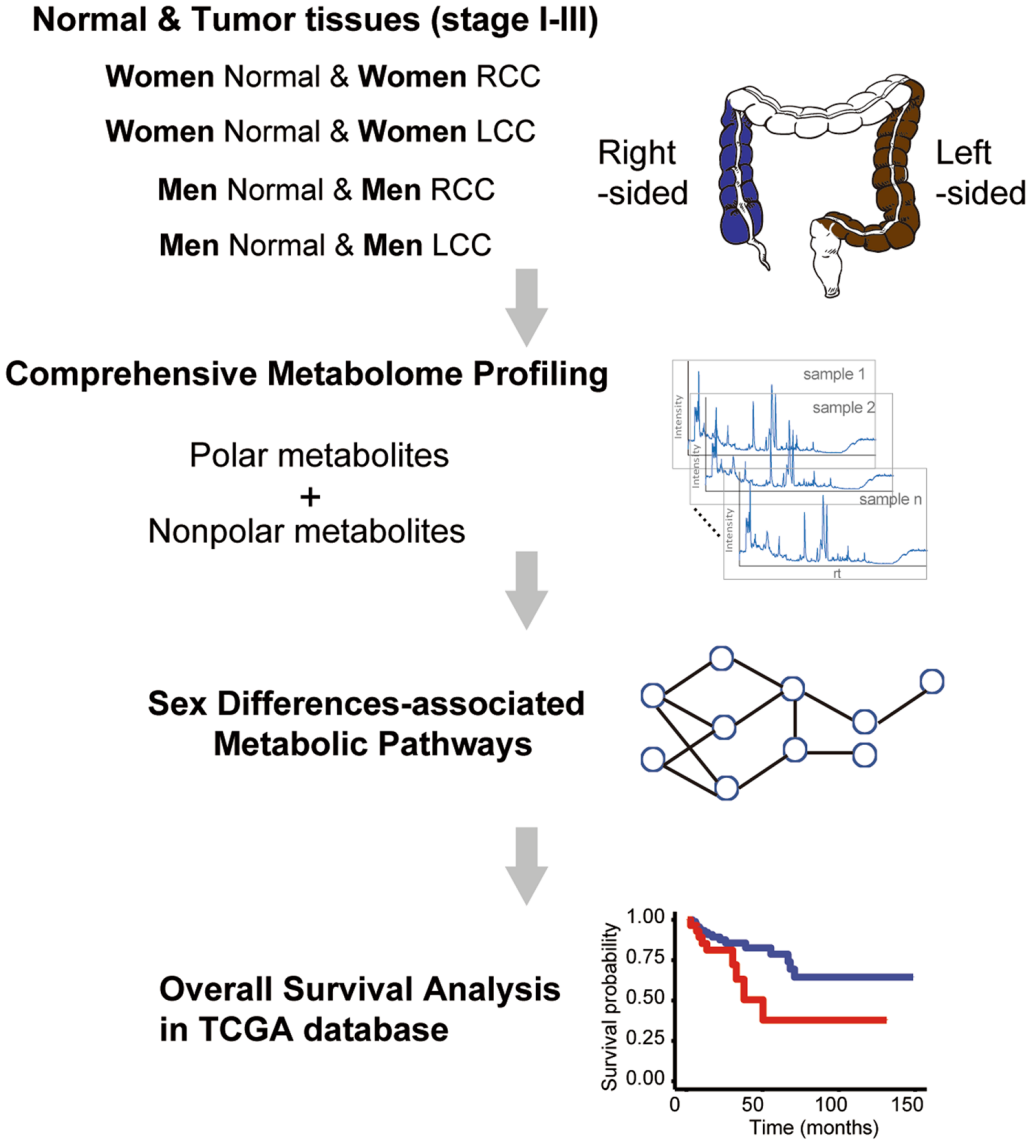
Schematic illustration of the metabolomics-driven analysis strategy for the discovery of sex-related differences in colon cancer metabolism. RCC, right-sided colon cancer; LCC, left-sided colon cancer; TCGA, The Cancer Genome Atlas.

Comprehensive untargeted metabolomics was carried out on normal colon and primary colon tumor tissues from a prospectively collected cohort of patients with RCC or LCC (n = 236). The detailed information of the clinical cohort is listed in Supplementary Table 1. Figure 1 illustrates the analysis strategy used within this study. Hydrophilic interaction liquid chromatography-mass spectrometry (HILIC-MS) and reversed-phase liquid chromatography (RPLC)-MS based metabolomics were carried out to measure the polar and nonpolar metabolome, respectively.

Initially, metabolite levels were compared between all normal colon and primary tumor tissues from men and women independently to identify tumor-specific metabolites for both sexes. The tumor metabolites were therefore normalized to controls within each sex to determine whether they are tumor specific versus a normal feature of colon tissue differences between men and women. Supplementary Table 2 shows the identified tissue metabolites and their statistical significance. When comparing normal colon tissues to tumors stratified by sex, the 82 metabolites that were altered in tumor tissues were represented in the following pathways: glycolysis, pentose phosphate pathway (PPP), amino acid metabolism, nucleotide metabolism, methionine metabolism, and lysophospholipid synthesis.

### Examination of sex and anatomic-specific differences in utilization of energy substrates

**Figure 2:**
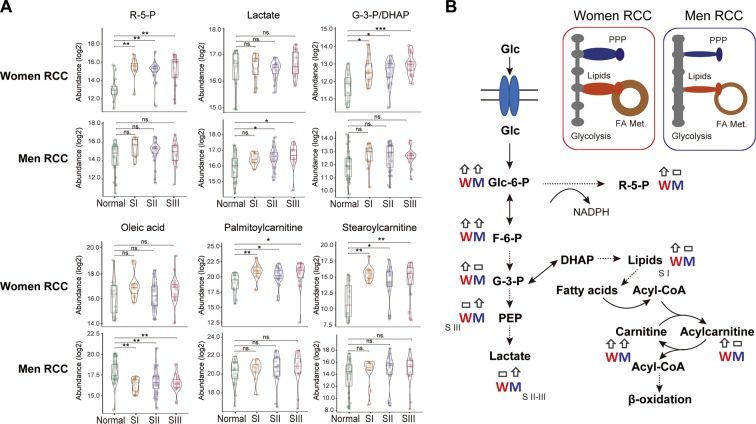
Sex differences in critical metabolites required for cancer cell growth. (**A**) ribulose-5-phosphate (R-5-P), glyceraldhyde-3-phosphate/dihydroxyacetone phosphate (G-3-P/DHAP), lactate, palmitoylcarnitine, stearoylcarnitine, and oleic acid in right-sided colon cancer (RCC). S = stage. Combination of box and violin plots display median value (center line), upper and lower quartiles (box limits), 1.5× interquartile range (black bar), and points out of interquartile range are outliers. Nonparametric Kruskal–Wallis rank sum test with pairwise Wilcoxon Mann-Whitney U test, *p* values adjusted for false discovery rates (FDR) (Benjamini-Hochberg). *p < 0.05, **p < 0.01, ***p < 0.001, ns. = not significant. Women RCC: Normal (n = 12), SI (n = 12), SII (n = 21), SIII (n = 17); Men RCC: Normal (n = 27), SI (n = 10), SII (n = 23), SIII (n = 15). (**B**) Schematic illustration of sex differences in glycolysis, pentose phosphate pathway (PPP), and fatty acid metabolism (FA Met.) in RCC. glucose (Glc), glucose-6-phosphate (Glc-6-P), fructose-6-phosphate (F-6-P), phosphoenolpyruvate (PEP). M = Men, W = Women. Up arrow indicates increased, horizontal rectangle indicates not changed in tumor compared to normal colon.

The initial analysis revealed sex-specific differences to metabolites that are critical end-products or intermediates in the PPP, glycolysis pathway, and the carnitine shuttle of fatty acids. We further examined the influence of both sex and anatomic location on the tumor metabolites. With an initial focus on patients with RCC, sex-specific differences were identified (Fig. 2). Ribulose-5-phosphate (R-5-P), an intermediate in the PPP that enables NADPH generation, was increased only in women with RCC across stages I-III (*p* < 0.01) but not in men with RCC. However, lactate, typically produced at high rates by cancer cells under a condition termed aerobic glycolysis^23^, was upregulated in men with RCC (stage II-III, *p* < 0.05) but not in women with RCC. Interestingly, the glycolysis intermediate glyceraldhyde-3-phosphate/dihydroxyacetone phosphate (G-3-P/DHAP) was only increased in women with RCC, and was upregulated across all stages (*p* < 0.05). Of note G-3-P and DHAP are isomers and could not be distinguished by mass spectrometry analysis, however, they are both important intermediates in the synthesis of lipids including lysophospholipids. The lysophospholipids lysophosphatidylcholine and lysophosphatidylethanolamine, were upregulated in women with RCC (stage I) but not in men (Supplementary Table 2). A recent study demonstrated that cancer cells can scavenge fatty acids from lysophospholipids to support cell growth^24^. Interestingly, in our cohort we found that oleic acid, a monounsaturated fatty acid (Fig. 2) was downregulated in men with RCC (*p* < 0.001), but its level was maintained in women with RCC, suggesting that the fatty acid supply was ensured by higher levels of lysophospholipids in women with RCC. In support of this hypothesis, increased fatty acyl carnitine levels (palmitoylcarnitine and stearoylcarnitine) were observed in women with RCC, indicative of enhanced fatty acid transportation via the carnitine shuttle, and oxidation to supply energy to tumors from women with RCC; this difference between controls and tumor samples was not observed in men.

For patients with LCC, similar differences in metabolites between normal and tumor tissues (R-5-P, lactate, oleic acid, palmitoylcarnitine, and stearoylcarnitine) were also observed (Supplementary Fig. 1). Lactate production was increased in men across all stages (*p* < 0.01), but not in women. The PPP and fatty acid metabolism was upregulated in women for all stages, *p* < 0.01 and *p* < 0.05 respectively. Subtle differences were seen for G-3-P/DHAP and R-5-P when comparing RCC and LCC for men. R-5-P was upregulated in LCCs stage I and G-3-P/DHAP upregulated LCCs stage I and II, and these metabolites were not increased in men with RCC, showing a possible utilization of both glycolysis and the PPP for men with LCC.

Combined, these results show a sex-specific difference in utilization of energy substrates in colon cancer. In women, this is independent of tumor location, however in men, some differences are evident between LCC and RCCs.

### Women with RCC have enhanced asparagine synthesis and amino acid uptake

**Figure 3:**
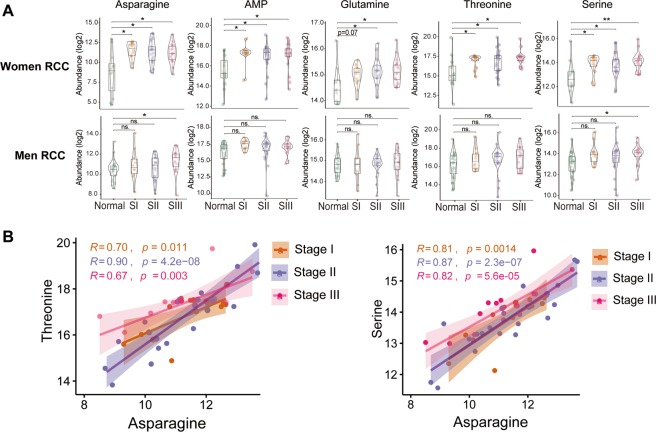
Increased asparagine synthesis associates with amino acid uptake in women with RCC. (**A**) Abundance of asparagine, adenosine monophosphate (AMP), glutamine, threonine, and serine in normal tissues and tumor tissues in stage I-III women with right-sided colon cancer (RCC) and men with RCC. Nonparametric Kruskal-Wallis rank sum test with pairwise Wilcoxon Mann-Whitney U test, *p* values adjusted for false discovery rate (FDR) (Benjamini-Hochberg). *p < 0.05, **p < 0.01, ***p < 0.001, ns. = not significant. Women RCC: Normal (n = 12), SI (n = 12), SII (n = 21), SIII (n = 17); Men RCC: Normal (n = 27), SI (n = 10), SII (n = 23), SIII (n = 15). (**B**) Pearson correlation analyses between asparagine with threonine, and serine in women with stage I-III RCC. Metabolite abundances were log2 transformed, and 95% confidence intervals (CIs) for the correlation coefficients were indicated as the shadowed area colored according to tumor stage.

Differences in amino acid metabolism were observed between tumors from both women and men when compared to controls. A further stratification by anatomic location revealed that asparagine synthesis, which is adenosine triphosphate (ATP)- and glutamine-dependent, was upregulated in women with RCC across all stages, (*p* < 0.05). However, asparagine was only upregulated in men with RCC at tumor stage III, (*p* < 0.05). Adenosine monophosphate (AMP) was increased only in women with RCC across all stages, (*p* < 0.05) (Fig. 3A) indicating increased hydrolysis of ATP to drive asparagine synthesis in both early and later stage tumors. In addition, glutamine was increased in women with RCC for both stage II and III tumors compared to normal colon (*p* < 0.05) which also supports increased asparagine synthesis. Glutamine was not increased in tumors from men with RCC.

As asparagine production has been previously linked to nutrient depletion and the uptake of additional amino acids^25^, we further investigated the differences in additional amino acid levels between men and women with RCC. As shown in Fig. 3A, threonine and serine were upregulated in women with RCC across stages I-III. Threonine was not changed in men with RCC at any stage, but serine was increased in stage III samples from men with RCC. We analyzed the metabolite-metabolite correlations across stage I-III samples from women with RCC to discover the association between asparagine and other metabolites related to this subset of colon cancer patients (Supplementary Fig. 2). Notably, asparagine levels were positively correlated with both threonine and serine at all tumor stages (Fig. 3B) (R > 0.6, *p* < 0.05, Pearson correlation). The increased uptake of amino acids, including serine in women with RCC, suggests increased nucleotide synthesis promoting cancer cell proliferation. Nucleotides such as guanosine monophosphate (GMP), deoxyinosine, uridine 5′-diphosphate (UDP) and the coenzyme flavin adenine dinucleotide (FAD) were upregulated in women with RCCs but no changes were present in tumors from men with RCC (Supplementary Table 2).

**Figure 4:**
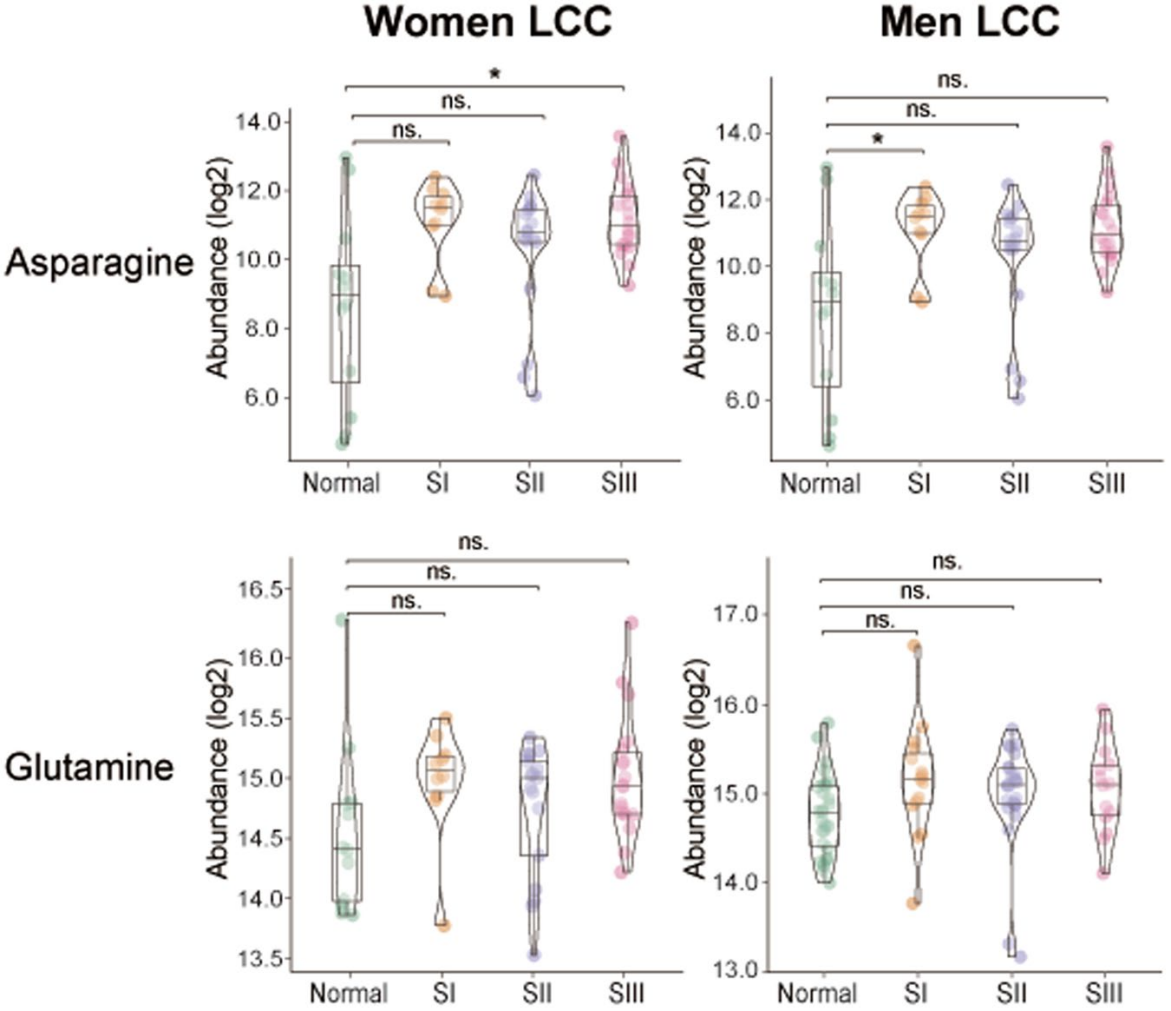
Abundance levels of asparagine and glutamine in normal and tumor tissues. Abundance levels are shown in normal tissue, stage I tumor, stage II tumor, and stage III tumors from women with left-sided colon cancer (LCC) and men with LCC. Nonparametric Kruskal–Wallis rank sum test with pairwise Wilcoxon Mann-Whitney U test, *p* values adjusted for false discovery rates (FDR) (Benjamini-Hochberg). *p < 0.05, **p < 0.01, ***p < 0.001, ns. = not significant. Women LCC: Normal (n = 12), SI (n = 10), SII (n = 17), SIII (n = 18); Men LCC: Normal (n = 27), SI (n = 15), SII (n = 25), SIII (n = 14).

In LCCs, asparagine was upregulated only in women with stage III and in men with stage I LCC (Fig. 4). Glutamine is an indispensable donor of nitrogen for the biosynthesis of asparagine catalyzed by asparagine synthetase (*ASNS*)^26^, however in LCCs, glutamine was not elevated in men or women with LCCs compared to control. Interestingly, the patterns of glutamine and asparagine between normal and LCCs across different stages in women are the same as in men with RCCs. However, serine, threonine and AMP are increased for women with stage III LCC, similar to the changes seen in these metabolites for women with RCC across all stages, but only serine is increased for men with stage I LCC (Supplementary Table 2).

Therefore, only tumors from women with RCC have enhanced asparagine synthesis supported by glutamine production, with concomitant increases in amino acid uptake and nucleotide production.

### High ASNS expression is associated with poorer overall survival for women with CRC

**Figure 5:**
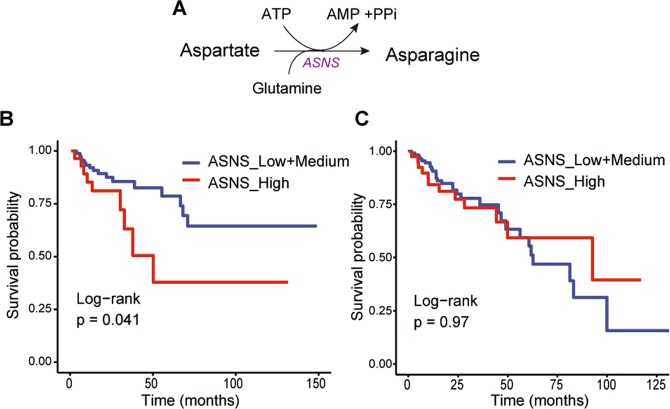
High asparagine synthetase (*ASNS*) expression was correlated with poorer overall survival for women with CRC. (**A**) Scheme of asparagine synthesis catalyzed by glutamine and ATP-dependent asparagine synthetase. Kaplan-Meier survival curve (overall survival probability) for (**B**) women with colon adenocarcinoma and (**C**) men with colon adenocarcinoma using data from The Cancer Genome Atlas (TCGA) database with low/medium and high *ASNS* expression levels.

The ATP and glutamine-dependent reaction, which converts aspartate to asparagine is catalyzed by ASNS (Fig. 5A). To test the association between *ASNS* expression and overall survival (OS) for women with colon cancer, and to validate our findings in an independent cohort, we carried out an analysis of TCGA gene expression data (RNA-Seq) deposited on cBioPortal^27,28^. This cohort contains *ASNS* gene expression data and clinical outcomes for 125 women with colon adenocarcinoma. Kaplan-Meier survival analysis (Fig. 5B), shows that high *ASNS* expression is associated with poorer OS in women with colon cancer (*p* = 0.041, log-rank test). However, *ASNS* does not affect OS in men with colon cancer (Fig. 5C). We also investigated the association of *ASNS* with OS for women and men stratified by anatomic location of the tumor. Interestingly, the results showed a trend between high *ASNS* expression and poorer OS for women with RCC in a limited sample size of patients (*p* = 0.062) (Supplementary Fig. 3). Our findings implicate a potential avenue for therapeutic intervention by regulating asparagine levels in colon cancer patients that have high *ASNS* expression.

To understand more closely the significance of identifying a subphenotype for women with RCC, based on our findings, we performed the first survival analysis on men and women by anatomic location of the tumor in the colon (LCCs and RCCs). Colon cancer patient data was derived from the SEER database (n = 267,472), and survival analysis showed that from 1990–2016, patients with RCC had poorer OS than those with LCC. This was seen for both women and men stages I-III, *p* < 0.0001 (Supplementary Fig. 4). The same differences were evident in each sex for each individual stage, *p* < 0.0001 (Supplementary Fig. 5). Moreover, the incidence of RCC in women was higher than in men (30.9 vs. 24.2 per 100,000 persons), while the incidence of LCC in women and in men was comparable (18.2 vs. 21.7 per 100,000 persons).

### Sex-related differences in methionine metabolism and polyamine synthesis pathways

**Figure 6:**
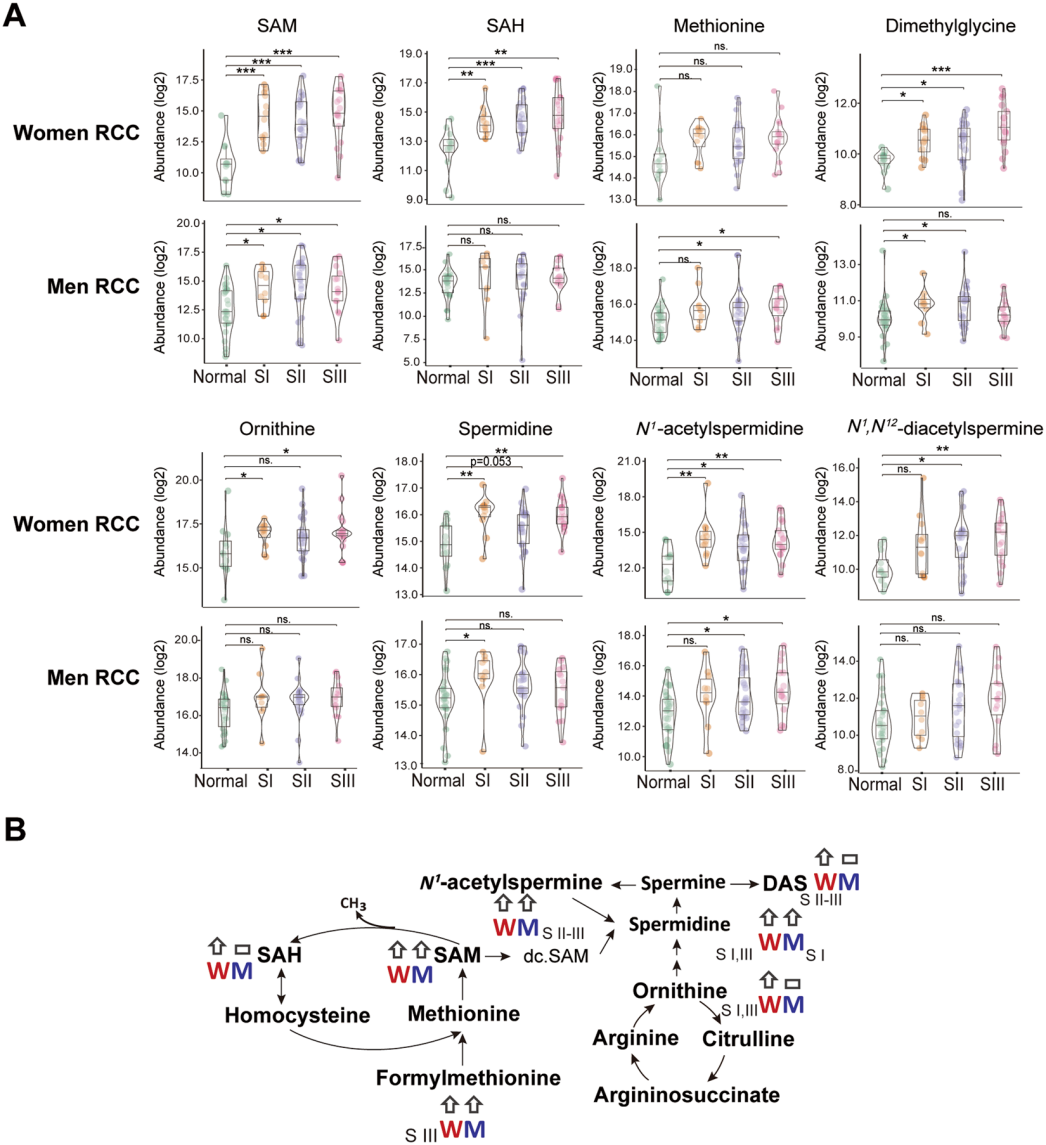
Sex-related differences in methionine and polyamine metabolism. (**A**) Abundance of S-adenosylmethionine (SAM), S-adenosylhomocysteine (SAH), methionine, dimethyglycine, ornithine, spermidine, *N*^1^-acetylspermidine, and *N*^1^, *N*^12^-diacetylspermine in right-sided colon cancer (RCC). S = stage. Nonparametric Kruskal–Wallis rank sum test with pairwise Wilcoxon Mann-Whitney U test, *p* values adjusted for false discovery rates (FDR) (Benjamini-Hochberg). *p < 0.05, **p < 0.01, ***p < 0.001, ns. = not significant. Women RCC: Normal (n = 12), SI (n = 12), SII (n = 21), SIII (n = 17); Men RCC: Normal (n = 27), SI (n = 10), SII (n = 23), SIII (n = 15). (**B**) Schematic illustration of sex differences in methionine metabolism and polyamine synthesis pathways in RCC. S-adenosylmethionine (SAM), decarboxylated S-adenosylmethionine (dc.SAM), S-adenosylhomocysteine (SAH), *N*^1^,*N*^12^-diacetylspermine (DAS). M = Men, W = Women, S = stage. Up arrow indicates increased, horizontal rectangle indicates not changed in tumor compared to normal colon.

In addition to sex-related differences in energy metabolism and asparagine synthesis, methionine and polyamine metabolic differences were also observed. In tumors from women with RCC, S-adenosylmethionine (SAM), S-adenosylhomocysteine (SAH), serine (a cofactor for SAH production), and dimethylglycine were upregulated across all stages when compared to normal colon tissue. Methionine, a precursor for SAM production was not changed. For men with RCC, SAM was upregulated across all stages, but there was no upregulation of SAH when compared to control tissue SAH levels (Fig. 6). Serine was only increased in stage III, and dimethylglycine was upregulated in stages I and II, however methionine was increased through stages II-III. Conversely for LCCs, men have SAM upregulated across all stages, and SAH was upregulated at stages I-II. For women, SAM, SAH, serine, were upregulated across all stages for LCCs, and dimethylglycine was upregulated at stages II-III (Supplementary Table 2). Therefore, for women anatomic differences in methionine metabolism were not seen.

One of the roles of SAM is to methylate polyamines, and increases were seen in multiple polyamines and their metabolites. *N*^1^, *N*^12^-diacetylspermine (DAS), was increased only in women with stage II-III RCCs, and it was not changed in men with RCCs. In tumors from women with RCC, spermidine was increased at stage I and III, and *N*^1^-acetylspermine increased across all stages, in men with RCC, tumors had increased spermidine at stage I, and *N*^1^-acetylspermine at stage II and III. Ornithine, a key metabolite in the urea cycle, is regulated by the tumor suppressor APC, and required for polyamine synthesis. Ornithine was upregulated in women with RCCs (stage I and stage III), similar to the changes seen for spermidine in women with RCC. Ornithine was not changed in men with RCC. For LCCs, DAS was increased across all stages for women, and not changed in men. Spermidine and *N*^1^-acetylspermine were both increased in stage III only in men, and increased in all stage tumors from women. Ornithine was not changed in any LCCs compared to normal tissues. Interestingly, formylmethionine, which is an initiator of protein synthesis, was upregulated in men at all stages for LCCs and RCCs whereas it was only increased in women at all stages for LCCs and advanced stage III for RCCs (Supplementary Table 2). Therefore, subtle polyamine differences exist by anatomic location, most notably for ornithine, whereas sex differences are shown for DAS and ornithine.

## Discussion

Colorectal cancer remains one of the leading causes of cancer related deaths in women and men worldwide. In this study, we identified significant sex-related differences in tissue metabolism for patients with RCC and LCC, with potential clinical implications. Metabolites in the glycolysis pathway, PPP, carnitine shuttle metabolism, asparagine synthesis, methionine metabolism, and the polyamine synthesis pathway all showed sex-related differences when measured in tumor samples compared to normal samples. We observed that tumors from women and men used different metabolic intermediates between the sexes for energy production to support cell growth. In tumors from women, products of glucose breakdown were shunted into the PPP, whereas in tumors from men, glucose was metabolized to primarily produce lactate. Furthermore, increased fatty acyl carnitine levels (palmitoylcarnitine and stearoylcarnitine) were observed in tumors from women. This is indicative of enhanced fatty acid transportation via the carnitine shuttle, and β-oxidation to generate energy. Fatty acid metabolism and oxidation produces more ATP than oxidation of glucose^29^, thus it is possible tumors from women require increased ATP for growth. Collectively, our results show women and men have different strategies to utilize substrates for energy production and tumor cell growth. For women with colon cancer, tumor cells supply energy to promote growth by utilizing oxidation of fatty acids, whereas glycolysis is increased in tumors from men. Further exploration by anatomic location revealed similar sex-specific differences in these pathways for RCCs, however men with LCC had some upregulated metabolites in the PPP in early stage tumors suggesting the utilization of both glycolytic metabolism and the PPP for these tumors.

Sex-associated differences in tumor metabolism by anatomic location were evident when examining asparagine production. Only women with RCC, had enhanced asparagine metabolism across all tumor stages. Asparagine is produced through a unidirectional ATP and glutamine-dependent reaction that converts aspartate to asparagine by *ASNS*, indicating that cells synthesize asparagine at the cost of cellular energy, and this reaction has been previously associated with tumor aggressiveness^30^. Previous studies have shown that *ASNS* is increased in high-grade anaplastic astrocytoma and glioblastoma, and correlates with poor prognosis for glioma and neuroblastoma patients^31^. We show for the first time, in an independent cohort, that high *ASNS* expression is associated with poorer OS in women with colon cancer but not in men. Further assessments of *ASNS* expression and its association with tumor location in the colon, show a trend of high expression and poorer OS in women with RCC. There was no such trend in *ASNS* expression and OS for LCC in both sexes or men with RCC. Therefore, asparagine metabolism could explain the link between tumor location difference and clinical outcomes in women with RCC compared to LCC^5,32^.

Studies have shown that asparagine can be used as an amino acid exchange factor^25^, the increase of glutamine, serine, and threonine serve as important sensors to control mechanistic target of rapamycin (mTOR) signaling that governs cell growth and autophagy^33^. Here we show that asparagine levels positively correlate with threonine and serine levels which were increased in women with RCC. Of note, our findings of these sex differences in metabolism are consistent with gene expression results on nutrient deplete conditions and the potential regulation of mTOR in women with RCC^34^. The increased uptake of amino acids can serve as substrates for nucleotide synthesis promoting cancer cell proliferation. Our study supports this in showing increased levels of nucleotides and FAD seen in women with RCCs only. Our data collectively suggests a mechanism by which asparagine increases threonine and serine uptake for tumor cell growth and survival in women with RCC.

**Figure 7:**
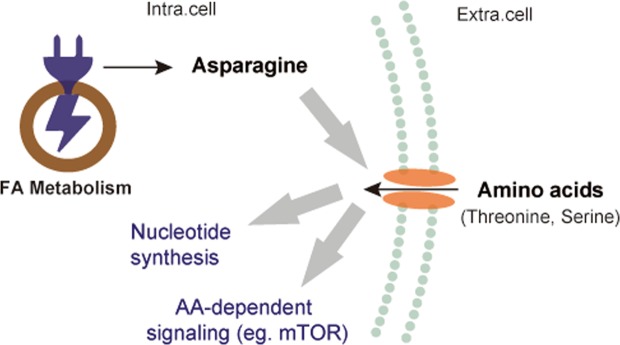
Proposed model of RCC metabolism in women. Enhanced fatty acid metabolism produces ATP required for asparagine production, which is associated with extracellular amino acid uptake. This promotes nucleotide synthesis and activates amino acid-dependent signaling. Fatty acid (FA), mammalian target of Rapamycin (mTOR), intracellular (Intra. cell), extracellular (Extra. cell), amino acid (AA).

We propose a model for women with RCC, whereby enhanced energy production is driven by fatty acid metabolism, which facilitates asparagine synthesis and amino acid uptake under nutrient-deplete conditions in the colon (Fig. 7). Asparagine therefore appears to play a critical role in maintaining tumor cell growth in women with RCC and could potentially affect tumor progression, aggressiveness and response to therapy. Asparagine is currently a therapeutic target for acute lymphocytic leukemia (ALL)^35^, where asparaginase drugs are used to decrease circulating asparagine levels. Asparaginase deprives cells of asparagine, which leads to cell death, and could be a potential valuable for treatment of women with colon cancer. A recent study carried out in SW480 and SW620 colon cancer cells lines, showed that chloroquine and asparaginase decrease asparagine levels, which subsequently retards cell growth and autophagy, and activates apoptosis^36^. A role for KRAS in asparagine metabolism was also suggested, and *ASNS* expression was shown to be induced by the PI3K-AKT-mTOR pathway and mutated KRAS^37^. This therefore opens another potential therapeutic avenue for patients with mutated KRAS who cannot receive anti-epidermal growth factor receptor-based therapies such as cetuximab and panitumumab^38^.

This study also revealed sex differences in methionine and polyamine metabolism in tumor tissues. Specifically, increased SAH and DAS were discovered only in women with RCCs but not in men with RCC. There are two plausible explanations for this. First, increased serine, dimethylglycine, SAH, and SAM indicates increased methionine cycling and potentially methylation in women with RCCs. This was also supported by increased polyamine methylation (DAS) seen in women with RCC. In men, SAM was increased in RCC, however SAH was not changed indicating that SAM is being shuttled for methylation purposes. For LCCs, SAM is upregulated across all stages for men, and SAH was upregulated at stages I-II. This indicates tumor location differences in methylation status for men. For women, these differences in SAM, SAH and serine, between LCCs and RCCs were not observed. These findings indicate perturbations to methionine cycling and potentially methylation in these patients. Unfortunately, it is not possible to determine the sex differences in methylation from this data. It has been previously observed that women were more likely to have methylation of the CpG island in the 5′ region of the *p16* ^INK4α^ tumor suppressor gene, compared to men. Moreover, higher levels of methylated tumors are noted to occur within RCCs, however for women we do not observe differences in methionine cycling between RCCs and LCCs^18^. A previous study that targeted the analysis of SAM and SAH in the blood plasma of CRC patients, indicated that in men, both higher SAM and SAH were associated with decreased risk of colorectal adenoma. In contrast, both higher SAM and SAH were associated with increased risk of colorectal adenomas in women^39^. Therefore, understanding these metabolites in the context of methylation will provide more understanding as to their effects on CRC development.

The treatment implications of polyamine synthesis are evident in the Southwest Oncology Group-National Cancer Institute sponsored NCT01349881 clinical trial examining the effects of the polyamine inhibitor eflornithine on reducing development of premalignant adenomatous polyps^40^. However, since the plasma metabolome encapsulates the systemic metabolome and multi-organ processing of metabolites, further studies in additional cohorts with clinical outcomes are necessary to validate this finding. Elevated DAS is also of interest in terms of CRC. This metabolite has been previously associated with multiple cancer types, including CRC, and is an end-product of polyamine metabolism. Our previous work showed that elevated DAS production in colon cancer was specific to tissues that had mucosal biofilms, which predominantly occurred on RCCs however, the sample size limitations did not allow for evaluation of sex-related differences^17,41^. However, it is possible that the microbiome plays a role in modulating metabolite production between men and women, as has been previously hypothesized^16,42^.

In addition to widespread differences between women and men, stage specific differences were seen. For example, lysophospholipids were only upregulated in stage I (node negative) tumors in women with RCC. Furthermore, in men with LCC, the metabolite G-3-P/DHAP was only upregulated in stage I and II (node negative) tumors, but not in advanced stage III (node positive) tumors. Our data suggests that sequential activation of metabolic pathways may exist for specific metabolites, and this may correlate with or drive colorectal cancer progression toward advanced stage disease. The progression of disease from stage II to stage III involves locoregional lymph node metastasis, which is a substantial change in cancer from being a process strictly localized in the colon to lymph (and then distant) metastasis. It is possible that lymph node metastasis is partly driven by lysophosphatidylethanolamine and G-3-P/DHAP, and these are necessary for development of metastasis. Additionally, lymph node metastasis may play a role in metabolites levels for advanced tumors. Whether or not these metabolites correlate with or drive metastasis needs further validation; it is the focus of experiments being tested in our lab to see if early stage biomarkers can be applied to clinical practice.

In conclusion, this study highlights the widespread metabolic differences are present among tissue specimens collected from men and women with CRC and the effect of anatomic location of the tumor. Notably, tumor tissue samples from women with RCC display enhanced energy production driven by fatty acid metabolism, which facilitates asparagine synthesis and a subsequent increase in amino acid uptake and nucleotide production. These processes together indicate nutrient-depletion in tumors in women with RCC. The metabolic differences observed in this study could result from not only the distinct presence of environmental factors, but also the influence of molecular features including microsatellite instability, mutations to MLH1 and BRAF, and CIMP when accounting for anatomic location in CRC. Future stratification by these molecular features could be valuable to uncover the link between sex differences in metabolism and molecular biology. As with all discovery metabolomics studies, further validation in an independent cohort would further support our findings. Our study also has potential implications in clinical management using precision medicine approaches, wherein targeting of asparagine levels for women with CRC are employed. This study therefore enhances our understanding of the mechanisms governing sex-specific differences in colon cancer metabolism.

## Materials and Methods

### Chemicals

Ammonium acetate and formic acid were purchased from Fisher Scientific (Morris Plains, NJ, United States). Ammonium hydroxide was purchased from Honeywell (Muskegon, MI, United States). Water (H_2_O), methanol (MeOH) and acetonitrile (ACN) were LC-MS grade and were purchased from Fisher Scientific (Morris Plains, NJ, United States).

### Sample collection

Colon tumor and normal colon tissue was acquired from surgery and prospectively collected on 736 stage I-IV CRC patients in the period 1991–2001 at Memorial Sloan-Kettering Cancer Center (MSKCC, New York, NY, United States). Clinical data was recorded and updated retrospectively. Tumor tissue and normal colon tissue (away from the tumor at the resection margin) was acquired from surgical colectomy specimens. Each sample was snap frozen in liquid nitrogen and immediately stored in a −80 °C freezer. Pre-operative intravenous antibiotics (cefazolin/metronidazole, clindamycin/gentamicin or ciprofloxacin/metronidazole) were administered within 60 min prior to resection. All patients received a standard mechanical bowel preparation (polyethylene glycol (PEG) solution) 24 h before scheduled surgery. For this study, samples were selected from patients that were ≥55 years old to reduce the confounding effects of estrogen signaling on metabolism before menopause. All normal colon tissues were selected from stage I-IV CRC patients (n = 39), and tumor tissue samples were selected from RCCs and LCCs stage I-III (n = 197). Stage IV tumor samples were not included as their metabolism may be affected by the presence of metastases in the liver or other site, therefore we cannot rule out this confounder. Normal samples were taken from stage IV patients, as their metabolic profiles were not significantly different from the normal colon tissues taken from stage I-III patients (Supplementary Fig. 6), and were markedly different from the tumor sample metabolomic profiles. The Yale University IRB determined that the study conducted in this publication was not considered Human Subjects Research and did not require IRB review (IRB/HSC# 1612018746). The study does not obtain data through intervention or interaction with the individual, or does not use or obtain identifiable private information. Informed consent was waived as part of the study exemption

### Tissue metabolite extraction

50 ± 1 mg of each tissue was homogenized using 500 μL of UPLC-grade H_2_O. A Cryolys Evolution homogenizer (Bertin Corporation, Rockville, MD, United States) was used with 2 mL lysing tube (Bertin Corporation) and 1.4 mm ceramic zirconium oxide beads (Bertin Corporation) to homogenize the tissues. Each sample was processed six times for 20 s, at 6,000 rpm with 5 s intervals. Dry ice was used to keep the temperature <10 °C during homogenization. From the homogenized solution, 100 μL was taken and added to 1.5 mL polypropylene microcentrifuge tubes for subsequent metabolite extraction. A volume of 400 μL ice cold MeOH:ACN (1:1, v/v) was added to each sample as the extraction solvent. The samples were vortexed for 30 s, and sonicated for 10 min. To precipitate proteins, the samples were incubated for 2 hours at −20 °C, followed by centrifugation at 13,000 rpm (15,000 g) and 4 °C for 15 min. The resulting supernatant was removed and evaporated to dryness for 12 hours using a vacuum concentrator (Thermo Fisher Scientific, Waltham, MA, United States). The dry extracts were then reconstituted in 100 µL of ACN:H_2_O (1:1, v/v), sonicated for 10 min, and centrifuged at 13,000 rpm (15,000 g) and 4 °C for 15 min to remove insoluble debris. The supernatant was transferred to UPLC autosampler vials (Thermo Scientific, MA, United States). A pooled quality control sample was prepared by mixing 5 μL of extracted solution from each sample into a UPLC autosampler vial. All the vials were capped and stored at −80 °C prior to UPLC-MS analysis.

### UPLC-MS analysis

Both HILIC-MS and RPLC-MS approaches were used for comprehensive analysis of the tissue metabolome. A UPLC system (H-Class ACQUITY, Waters Corporation, MA, United States) coupled to a quadrupole time-of flight (QTOF) mass spectrometer (Xevo G2-XS QTOF, Waters Corporation, MA, United States) was used for MS data acquisition. A Waters ACQUITY UPLC BEH Amide column (particle size, 1.7 μm; 100 mm (length) × 2.1 mm (i.d.)) and Waters ACQUITY UPLC BEH C18 column (particle size, 1.7 μm; 50 mm (length) × 2.1 mm (i.d.)) were used for the UPLC-based separation of metabolites. The column temperature was kept at 25 °C for HILIC-MS analysis and 30 °C for RPLC-MS analysis. The solvent flow rate was 0.5 mL/min, and the sample injection volume was 1 μL. For HILIC-MS analysis, mobile phase A was 25 mM NH_4_OH and 25 mM NH_4_OAc in water, while the mobile phase B was ACN for both electrospray ionization (ESI) positive and negative mode, respectively. The linear gradient was set as follows: 0~0.5 min: 95% B; 0.5~7 min: 95% B to 65% B; 7~8 min: 65% B to 40% B; 8~9 min: 40% B; 9~9.1 min: 40% B to 95% B; 9.1~12 min: 95% B. For RPLC-MS analysis, the mobile phases A was 0.1% formic acid in H_2_O, while the mobile phases B was 0.1% formic acid in ACN, respectively for both ESI+ and ESI−. The linear gradient was set as follows: 0~1 min: 1% B; 1~8 min: 1% B to 100% B; 8~10 min: 100% B; 10~10.1 min: 100% B to 1% B; 10.1~12 min: 1% B. Pooled samples were analyzed every eight injections during the UPLC-MS analysis to monitor the stability of the data acquisition and used for subsequent data normalization.

QTOF-MS scan data (300 ms/scan; mass scan range 50–1000 Da) was initially acquired for each biological sample for metabolite quantification. Then, both DDA (data-dependent acquisition) data (QTOF MS scan time: 50 ms/scan, MSMS scan time 50 ms/scan, collision energy 20 eV, top 5 most intense ions were selected for fragmentation, exclude former target ions (4 s after 2 occurrences)) and MS^e^ data (low energy scan: 200 ms/scan, collision energy 6 eV; high energy scan: 100 ms/scan, collision energy 20 eV, mass scan range 25–1000 Da) were acquired for QC samples to enable metabolite identification. ESI source parameters on the Xevo GS-XS QTOF were set as following: capillary voltage 1.8 kV, sampling cone 40 V, source temperature 50 °C, desolvation temperature 550 °C, cone gas flow 40 L/Hr, desolvation gas flow 900 L/Hr.

### UPLC-MS data processing

The raw MS data (.raw) were converted to mzML files using ProteoWizard MSConvert^43^ (version 3.0.6150, http://proteowizard.sourceforge.net/). The files were then processed in R (version 3.4.3) using the XCMS package^44^ for feature detection, retention time correction and alignment. The XCMS processing parameters were optimized and set as follows: mass accuracy for peak detection = 25 ppm; peak width c = (2, 30); snthresh = 6; bw = 10; mzwid = 0.015; minfrac = 0.5. The CAMERA package^45^ was used for subsequent peak annotation. The resulting data were normalized using the support vector regression algorithm in R to remove unwanted system error that occurred among intra- and inter- batches^46^. Initial metabolite identification was performed using the MetDNA algorithm (metabolomics standard initiative levels^47^ can be seen in Supplementary Table 2^48^). Metabolites were further identified by matching retention time with an in-house metabolite standard library. In addition, metabolite identification was carried out by matching accurate mass and experimental MS/MS data against online databases (METLIN^49^ and HMDB^50^). The detailed identification information and identification confidence levels of dysregulated metabolites are listed in Supplementary Table 2.

### Statistical analysis

Statistical analyses were performed in R (version 3.4.3). Nonparametric Kruskal–Wallis rank sum test with pairwise Wilcoxon Mann-Whitney U test were applied. *p* values were adjusted for multiple testing with Benjamini-Hochberg-based FDR. Pearson correlation analyses between metabolites were also performed using package “ggpubr” in R.

Survival analyses on colon cancer patients (n = 267,472) from SEER database were carried out to investigate outcomes between patients with RCC and patients with LCC. All data were downloaded using SEER*Stat software (version 8.3.5, https://seer.cancer.gov/seerstat/). Patients age 55 years and older who were diagnosed with primary adenocarcinoma of the colon from 1990 to 2016 were included in the analysis if they had a diagnosis of American Joint Committee on Cancer (AJCC) stage I to III colon adenocarcinoma. Tumor location within the colorectum was designated right-sided (C18.0, C18.2, and C18.3, corresponding to cecum, ascending colon, and hepatic flexure) or left-sided (C18.5, C18.6, C18.7, C19.9, corresponding to splenic flexure, descending colon, sigmoid colon, and rectosigmoid junction) according to the *International Classification of Diseases for Oncology*, 3rd edition (ICD-03). Patients with rectal, appendiceal, and transverse primary tumors were excluded. Kaplan-Meier survival analyses were conducted in R programming language using R packages “survminer” and “survival”. Log-rank test was utilized to analyze the statistical differences between different groups of colon cancer patients.

Comparative analysis on incidences for women and men stratified with tumor location was performed on colon cancer patients (age ≥ 55 years old) recorded from 1992 to 2016. Data was extracted using SEER*Stat software (version 8.3.5). Patients with rectal, appendix, and transverse primary tumors were excluded. The incidence rates (using the 2000 U.S. standard population) express per 100,000 persons.

In order to assess the correlation of our findings to patient outcomes we analyzed TCGA colon cancer data set (TCGA Provisional). This was downloaded through cBioPortal v2.2.1^27,28^. Z-Scores (RNA Seq V2 RSEM) were used as a measure of mRNA expression. In addition to gene expression data, patient data including clinical parameters such as sex, age, survival status, and OS time were also downloaded. Patients with the colon cancer type colon adenocarcinoma (COAD) were selected. OS analyses were carried out only on patients with available survival and mRNA expression data. Two groups of samples: (a) High *ASNS* expression (with Z-Scores above upper quartile) and (b) Low *ASNS* expression (with Z-Scores equal or below upper quartile) were categorized as previously described^51^. Kaplan-Meier survival analyses were conducted in R programming language using R packages “survminer” and “survival”. Log-rank test was utilized to analyze the statistical differences between different groups of colon cancer patients.

## Supplementary information

Supplementary Information file.

Supplementary Information Table S2.
