# Supplementary material for: Sex Differences in Colon Cancer Metabolism Reveal A Novel Subphenotype
Source: Sci Rep. 2020 Mar 17;10:4905. doi: 10.1038/s41598-020-61851-0 (PMC7078199; doi:10.1038/s41598-020-61851-0)
Supplement: Supplementary file 1 — Supplementary Information file. [file 41598_2020_61851_MOESM1_ESM.docx]

***Supplementary Information for***

**Sex Differences in Colon Cancer Metabolism Reveal A Novel Subphenotype**

**Authors**

Yuping Cai, Nicholas J.W. Rattray, Qian Zhang, Varvara Mironova, Alvaro Santos-Neto, Kuo-Shun Hsu, Zahra Rattray, Justin R. Cross, Yawei Zhang, Philip B. Paty, Sajid A. Khan, Caroline H. Johnson

Sajid A. Khan, Department of Surgery, Division of Surgical Oncology, Yale University School of Medicine, New Haven, CT, USA.

[sajid.khan@yale.edu](mailto:sajid.khan@yale.edu)

Caroline H. Johnson, Department of Environmental Health Sciences, Yale School of Public Health, Yale University, New Haven, CT USA

[caroline.johnson@yale.edu](mailto:caroline.johnson@yale.edu)

|  | **Normal (n=39)** | | **Stage I (n=47)** | | **Stage II (n=86)** | | | **Stage III (n=64)** | | |
| --- | --- | --- | --- | --- | --- | --- | --- | --- | --- | --- |
|  |  |  | **RCC**  **(n=22)** | **LCC**  **(n=25)** | | **RCC**  **(n=44)** | **LCC**  **(n=42)** | | **RCC**  **(n=32)** | **LCC**  **(n=32)** |
| **Sex, n** |  |  | |  | |  |  | |  |  |
| Male | 27 | | 10 | 15 | | 23 | 25 | | 15 | 14 |
| Female | 12 | | 12 | 10 | | 21 | 17 | | 17 | 18 |
| **Age, mean (sd)** | | | | | | | | | | |
| Male | 69.3 (9.6) | | 73.9 (6.5) | 69.3 (5.8) | | 72.9 (7.8) | 72.2 (8.5) | | 73.5 (7.8) | 63.7 (5.8) |
| Female | 63.3 (16.1) | | 72.1 (6.2) | 69.6 (7.6) | | 73.5 (9.8) | 69.1 (7.8) | | 72.2 (6.6) | 71.1 (6.0) |
| **Race/Ethnicity, n** | | | | | | | | | | |
| NHWs | 35 | | 20 | 22 | | 40 | 37 | | 26 | 25 |
| Hispanic | 2 | | 2 | 3 | | 2 | 3 | | 4 | 2 |
| AA | 1 | | 0 | 0 | | 1 | 0 | | 1 | 4 |
| API | 1 | | 0 | 0 | | 1 | 2 | | 1 | 1 |
| NHWs: non-Hispanic whites, AA: African-Americans, API: Asian-Pacific Islander | | | | | | | | | | |

**Table S1**. Demographics of colon cancer patients from samples used within this study. Right-sided colon cancer = RCC, Left-sided colon cancer = LCC.


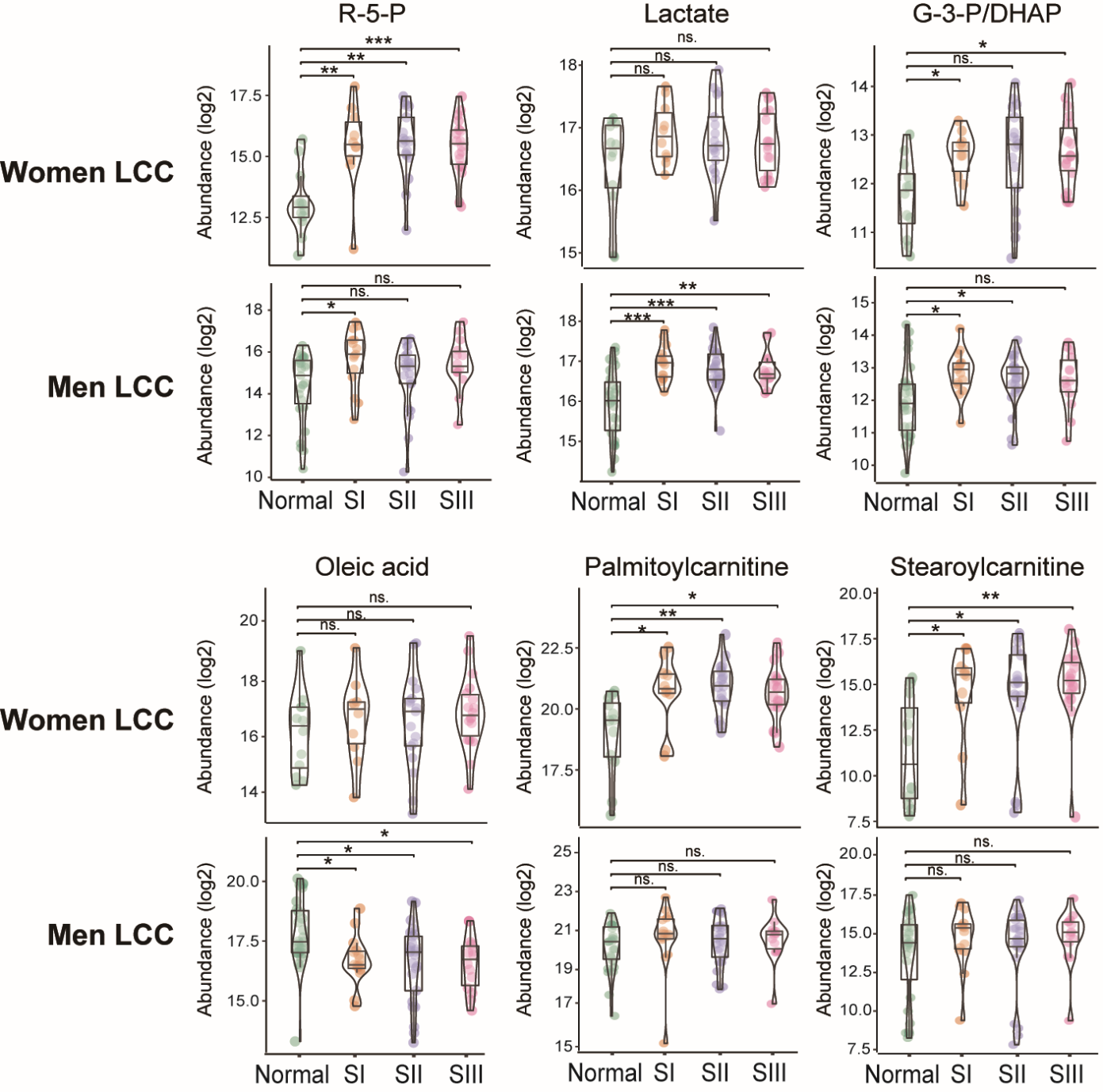


**Fig. 1**: Sex differences in critical metabolites including R-5-P (ribulose-5-phosphate), G-3-P/DHAP (glyceraldhyde-3-phosphate/dihydroxyacetone phosphate), lactate, palmitoylcarnitine, stearoylcarnitine, and oleic acid in left-sided colon cancer (LCC). S=stage. Nonparametric Kruskal–Wallis rank sum test with pairwise Wilcoxon Mann-Whitney U test, *p* values adjusted for false discovery rates (FDR) (Benjamini-Hochberg). **p*<0.05, ***p*<0.01, ****p*<0.001, ns. = not significant. Women LCC: Normal (n=12), SI (n=10), SII (n=17), SIII (n=18); Men LCC: Normal (n=27), SI (n=15), SII (n=25), SIII (n=14).


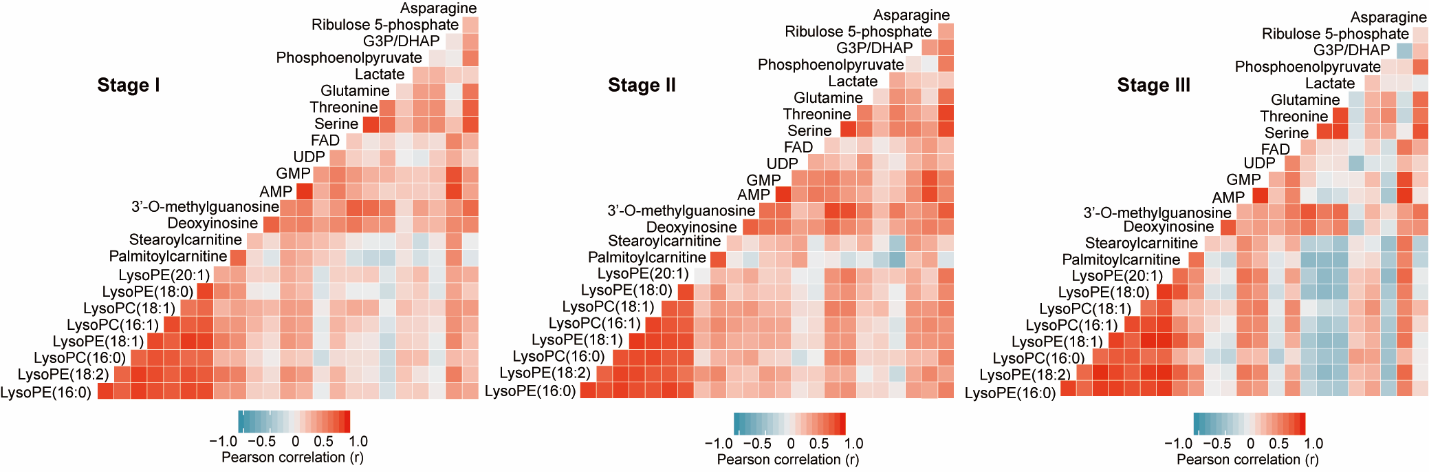


**Fig. 2**: Heatmap of metabolite-metabolite correlations in women with right-sided colon cancer across stages I-III. Pearson correlation coefficients were calculated. Positive correlations are shown as red square box, and negative correlations are shown as blue square box.


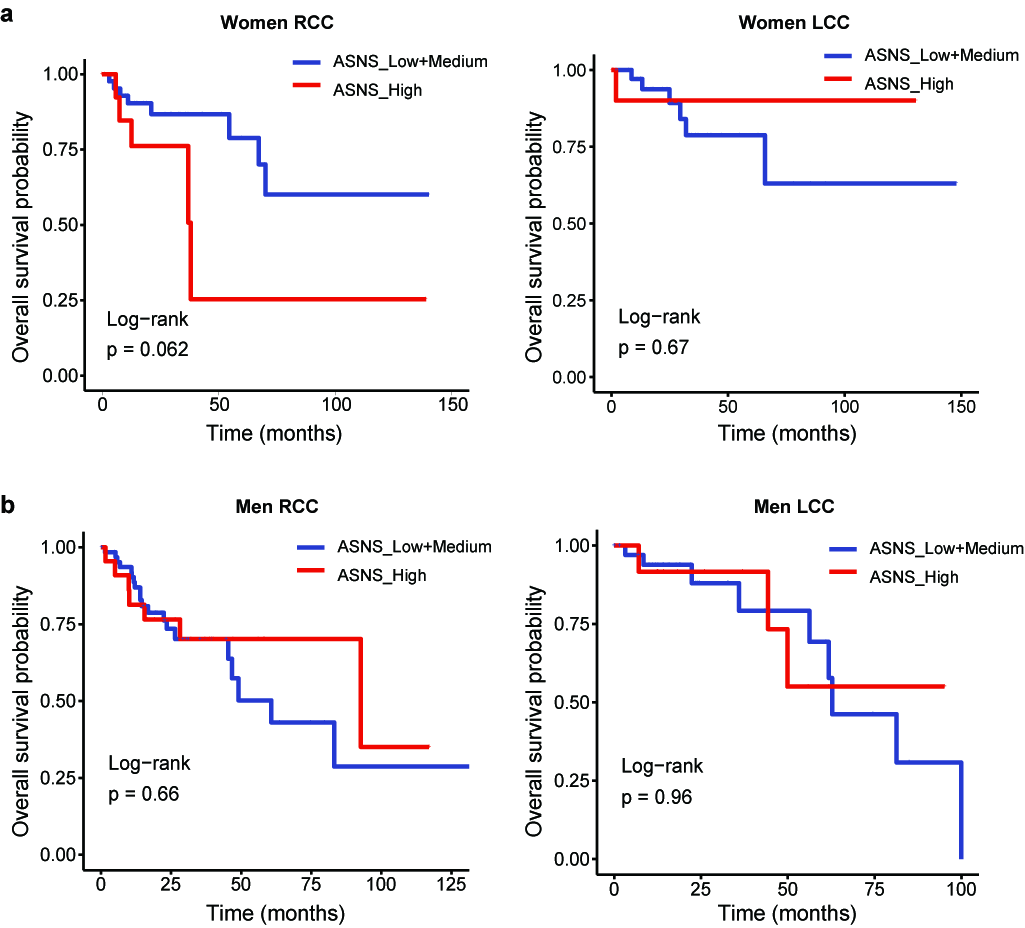


**Fig. 3**: Kaplan-Meier survival curve (overall survival) for patients with colon adenocarcinoma using data from The Cancer Genome Atlas (TCGA) database with low/medium and high ASNS expression levels, respectively. **a** Women with RCC and LCC. **b** Men with RCC and LCC.


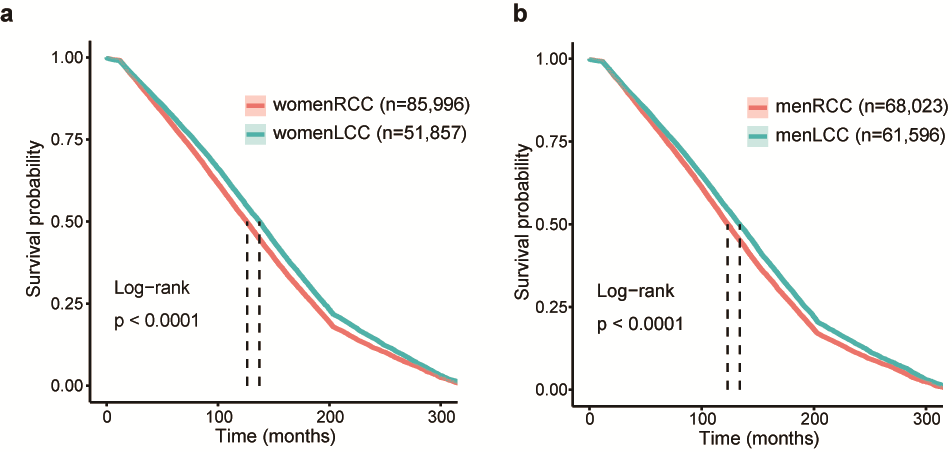


**Fig. 4**: Kaplan-Meier curves depicting overall survival for patients with colon cancer (from 1990 to 2016, age>=55 years) using data from the Surveillance, Epidemiology, and End Results (SEER) database stratified by primary tumor location (stages I-III). **a**. Comparison between women with RCC and LCC. **b**. Comparison between men with RCC and LCC.


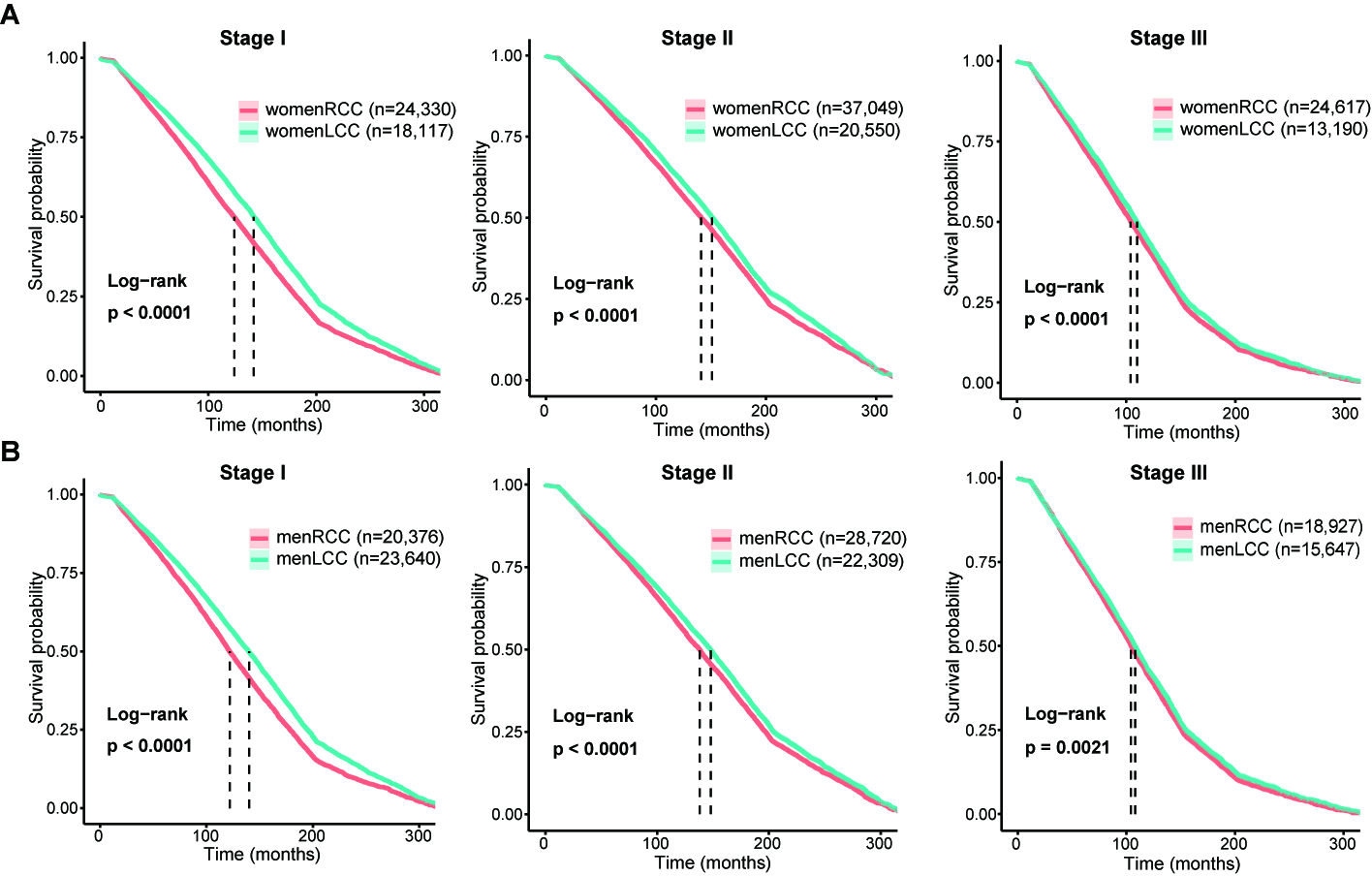


**Fig. 5**: Kaplan-Meier curves depicting overall survival for (**A**) women with colon cancer and (**B**) with colon cancer (from 1990 to 2016) using data from the Surveillance, Epidemiology, and End Results (SEER) database stratified by primary tumor location and stage. SEER database accessed July 20, 2019. Patients met the following criteria: age>=55 years, stages I-III; exclusion of rectal, appendiceal, and transverse primary tumors. Right-sided colon cancer = RCC, Left-sided colon cancer = LCC.


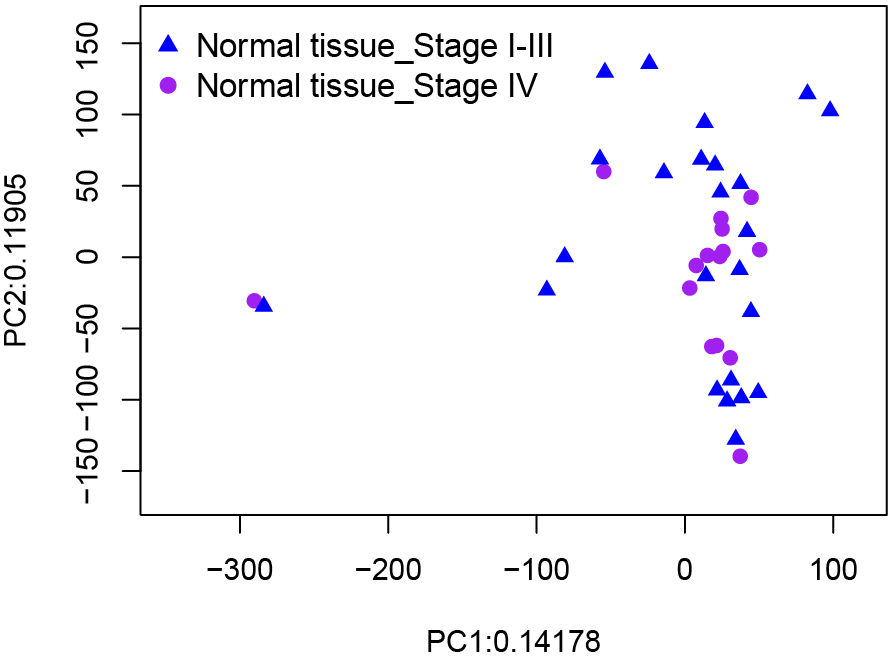


**Fig. 6**: PCA scores plot on normal tissue from stage I-III patients and stage IV patients.
