# Supplementary material for: Sex Differences in Colon Cancer Metabolism Reveal A Novel Subphenotype
Source: Sci Rep. 2020 Mar 17;10:4905. doi: 10.1038/s41598-020-61851-0 (PMC7078199; doi:10.1038/s41598-020-61851-0)
Supplement: Supplementary file 2 — Supplementary Information Table S2. [file 41598_2020_61851_MOESM2_ESM.pdf]

Supplementary Information Table S2: Identified tissue metabolites significantly changed in colon cancer tumor stratified by sex, anatomic location, and stage. (Yellow highlighted content represents p<0.05)

	womenRCC						menRCC					
	Fold change			p value			Fold change			p value		
Metabolite	S I/Normal	S II/Normal	S III/Normal	S I/Normal	S II/Normal	S III/Normal	S I/Normal	S II/Normal	S III/Normal	S I/Normal	S II/Normal	S III/Normal
Glutamate	1.10	1.09	1.10	7.27E-03	5.30E-03	5.30E-03	1.06	1.07	1.06	2.77E-03	1.77E-03	1.98E-03
Glutamine	1.05	1.05	1.05	7.74E-02	1.61E-02	1.61E-02	1.00	1.01	1.01	9.06E-01	9.06E-01	9.06E-01
Aspartate	1.16	1.16	1.15	3.82E-04	3.04E-03	3.82E-04	1.11	1.10	1.11	2.06E-02	2.06E-02	7.92E-02
Asparagine	1.32	1.29	1.24	1.66E-02	1.66E-02	1.66E-02	1.02	1.04	1.11	4.45E-01	3.92E-01	3.50E-02
Dimethylglycine	1.07	1.09	1.12	1.67E-02	2.25E-02	3.59E-04	1.09	1.10	1.03	4.45E-02	4.45E-02	2.89E-01
Serine	1.12	1.08	1.12	2.00E-02	2.00E-02	9.40E-03	1.05	1.04	1.07	8.09E-02	8.09E-02	2.03E-02
Uracil	1.07	1.07	1.07	1.04E-01	3.00E-02	2.61E-02	1.07	1.07	1.06	5.80E-04	8.52E-05	5.21E-05
Creatinine	0.93	0.96	0.95	8.98E-02	7.08E-02	7.08E-02	0.95	0.94	0.93	4.70E-02	5.97E-02	4.70E-02
succinate	1.02	1.00	1.02	9.77E-01	9.77E-01	9.77E-01	1.01	1.02	1.01	5.25E-01	5.25E-01	5.25E-01
Threonine	1.15	1.15	1.16	4.87E-02	4.87E-02	1.59E-02	1.01	1.04	1.05	7.06E-01	1.97E-01	1.97E-01
Hypoxanthine	1.04	1.04	1.05	1.39E-02	1.39E-02	3.59E-04	1.04	1.02	1.03	1.43E-03	9.17E-02	1.43E-03
Taurine	1.08	1.08	1.08	1.57E-06	1.57E-06	2.31E-07	1.07	1.07	1.07	4.88E-04	4.70E-05	4.16E-04
Ornithine	1.08	1.06	1.07	3.68E-02	1.50E-01	3.68E-02	1.03	1.03	1.03	1.95E-01	1.27E-01	1.27E-01
Histidine	1.14	1.17	1.15	7.26E-02	7.28E-02	5.55E-02	1.04	1.06	1.06	1.16E-01	5.94E-02	1.16E-01
Citrulline	1.09	1.08	1.15	3.37E-01	3.37E-01	1.10E-01	1.09	1.05	1.04	2.13E-02	1.66E-01	1.54E-01
Lysine	1.07	1.07	1.08	2.01E-01	2.01E-01	3.05E-02	1.03	1.02	1.03	2.86E-01	2.86E-01	2.86E-01
Alanine	1.21	1.12	1.16	9.03E-03	7.96E-03	7.96E-03	1.16	1.10	1.14	4.80E-04	3.59E-03	1.26E-03
Methionine	1.10	1.05	1.09	5.98E-02	5.98E-02	5.98E-02	1.04	1.05	1.05	9.61E-02	3.98E-02	3.98E-02
Adenine	1.06	1.09	1.07	1.45E-01	7.58E-02	1.45E-01	1.07	1.05	1.03	6.20E-02	6.20E-02	2.86E-01
N-Formylmethionine	1.06	1.05	1.15	1.36E-01	9.50E-02	2.93E-03	1.04	1.08	1.02	3.43E-02	1.14E-05	3.92E-02
Acetyl-lysine	1.45	1.29	1.44	3.01E-02	5.40E-02	1.59E-02	1.23	1.21	1.18	9.37E-03	9.37E-03	4.54E-02
NG,NG-dimethyl-L-arginine (ADMA)	1.16	1.10	1.16	2.49E-02	4.39E-02	4.40E-03	1.07	1.06	1.11	1.03E-03	1.83E-03	1.58E-03
Argininosuccinic acid	1.28	1.24	1.34	2.49E-02	1.38E-01	1.34E-02	1.16	1.08	1.11	9.57E-03	3.54E-02	5.63E-02
Glucose 6-phosphate	1.12	1.11	1.14	3.45E-02	3.37E-02	4.40E-03	1.10	1.09	1.06	2.87E-03	5.38E-03	4.88E-02
Fructose 6-phosphate	1.25	1.23	1.24	2.88E-04	1.43E-04	2.32E-04	1.25	1.22	1.21	1.31E-03	6.82E-04	6.82E-04
Phosphoenolpyruvate	1.12	1.04	1.07	9.31E-01	2.66E-01	2.66E-01	1.12	1.03	1.06	7.70E-02	2.71E-01	3.82E-02
DHAP/G3P	1.05	1.08	1.09	2.90E-02	1.41E-02	7.64E-04	1.09	1.09	1.07	1.95E-01	1.53E-01	1.18E-01
Lactate	1.00	0.99	0.99	9.77E-01	8.53E-01	8.53E-01	1.03	1.04	1.04	1.56E-01	4.98E-02	2.68E-02
Ribulose 5-phosphate	1.21	1.19	1.24	3.13E-03	3.13E-03	3.13E-03	1.06	1.03	1.01	2.22E-01	4.33E-01	5.42E-01
Glucosamine 6-phosphate	1.14	1.11	1.11	1.75E-02	3.66E-02	3.66E-02	1.17	1.11	1.09	3.91E-02	3.98E-02	4.54E-02
S-Adenosylhomocysteine	1.11	1.13	1.16	1.97E-03	6.61E-04	3.75E-03	1.11	1.05	1.02	4.21E-01	4.21E-01	4.21E-01
S-Adenosylmethionine	1.36	1.30	1.38	9.91E-05	8.77E-05	9.91E-05	1.18	1.23	1.14	4.23E-02	1.33E-02	4.23E-02
Spermidine	1.09	1.05	1.07	2.57E-03	5.40E-02	2.57E-03	1.05	1.02	1.02	4.39E-02	7.67E-02	5.23E-01
Acetylspermine	1.17	1.12	1.14	2.57E-03	3.94E-02	2.57E-03	1.09	1.04	1.09	8.83E-02	4.71E-02	3.50E-02
Diacetylspermine	1.15	1.22	1.24	1.56E-01	1.61E-02	9.40E-03	1.05	1.10	1.14	5.50E-01	3.07E-01	1.46E-01
Glycerol 3-phosphate	1.01	0.97	0.97	5.65E-01	5.65E-01	5.65E-01	1.04	0.96	0.98	5.55E-01	1.46E-01	3.94E-01
Palmitic acid	1.10	1.08	1.10	4.98E-02	5.42E-01	7.61E-02	0.99	0.99	0.99	8.04E-01	7.52E-01	7.52E-01
Oleic acid	1.03	0.99	1.03	3.04E-01	7.26E-01	4.55E-01	0.95	0.95	0.94	1.43E-03	7.19E-03	1.43E-03
Stearic acid	1.05	1.04	1.05	5.66E-01	5.66E-01	5.66E-01	1.07	1.08	1.04	1.81E-01	6.54E-02	2.93E-02
Carnitine	1.02	1.02	1.02	3.82E-04	8.25E-03	3.82E-04	1.01	1.01	1.02	6.30E-03	1.98E-02	1.56E-03
Palmitoylcarnitine	1.07	1.03	1.08	6.69E-03	4.87E-02	2.07E-02	1.01	1.02	1.02	6.91E-01	4.53E-01	4.53E-01
Stearoylcarnitine	1.47	1.34	1.46	1.21E-03	2.53E-02	9.41E-03	1.05	1.08	1.06	7.06E-01	7.06E-01	7.06E-01
LysoPE(16:1)	1.12	1.05	1.06	5.15E-03	1.23E-01	5.49E-02	1.12	1.07	1.06	5.35E-03	4.54E-02	4.54E-02
LysoPE(16:0)	1.04	1.00	1.01	1.75E-02	5.65E-01	4.87E-01	1.02	1.00	0.98	5.22E-01	6.25E-01	7.03E-01
LysoPE(18:2)	1.05	1.01	1.04	6.69E-03	3.26E-01	6.02E-02	1.02	1.01	1.01	6.94E-01	6.94E-01	8.15E-01
LysoPE(18:1)	1.06	1.01	1.03	6.69E-03	2.24E-01	7.96E-02	1.02	1.00	0.98	3.86E-01	8.36E-01	8.36E-01
LysoPC(16:1)	1.07	1.02	1.07	3.34E-02	4.27E-01	1.31E-01	1.03	1.01	0.98	3.24E-03	7.90E-01	9.17E-01
LysoPC(18:1)	1.09	1.04	1.05	5.15E-03	2.68E-01	9.52E-02	1.03	0.98	1.01	7.00E-02	6.02E-01	6.02E-01
LysoPE(18:0)	1.06	1.02	1.02	1.11E-02	4.38E-01	4.24E-01	1.05	1.02	1.03	4.65E-01	9.06E-01	9.06E-01
LysoPE(20:1)	1.12	1.06	1.07	6.69E-03	1.50E-01	2.40E-02	1.07	1.04	1.03	1.40E-01	5.41E-01	5.41E-01
LysoPE(22:5)	1.02	0.97	0.95	2.05E-01	8.68E-01	7.76E-01	1.01	0.97	0.99	2.30E-01	2.13E-01	2.30E-01
PE(34:1)	1.02	1.08	1.01	3.62E-01	3.37E-02	5.86E-01	1.05	1.08	1.12	1.68E-01	2.63E-02	1.76E-03
PE(36:1)	1.01	1.05	0.97	9.32E-01	3.26E-01	8.15E-01	1.00	1.05	1.05	9.06E-01	9.05E-02	9.05E-02
O-Phosphoethanolamine	1.18	1.15	1.15	4.33E-04	1.38E-03	4.33E-04	1.13	1.08	1.11	3.20E-04	4.13E-02	8.80E-04
Stearoylethanolamide	1.06	1.01	1.02	3.34E-02	5.65E-01	3.95E-01	1.04	0.99	0.99	4.33E-01	7.12E-01	9.30E-01
Glucuronate	1.13	1.09	1.02	9.97E-02	4.38E-01	8.79E-01	0.97	0.95	0.97	8.56E-01	4.65E-01	8.56E-01
Sphinganine-1-phosphate	1.05	1.00	1.01	1.75E-02	6.22E-01	4.87E-01	1.05	1.00	1.00	8.26E-02	9.30E-01	9.30E-01
Pantothenate	1.08	1.10	1.09	8.82E-02	8.82E-02	8.82E-02	1.13	1.06	1.06	9.57E-03	3.92E-02	3.43E-01
Vitamin E	1.13	1.15	1.11	1.79E-01	1.79E-01	1.95E-01	0.94	1.01	0.98	6.77E-01	6.77E-01	9.79E-01
Cytidine	1.06	1.02	1.01	9.48E-01	9.48E-01	9.48E-01	0.96	0.96	0.87	3.64E-01	1.89E-01	1.14E-02
Deoxyinosine	1.23	1.18	1.18	8.74E-03	1.77E-02	8.74E-03	1.12	1.11	1.12	1.48E-01	1.48E-01	1.48E-01
Adenosine	0.99	1.00	1.03	1.00E+00	1.00E+00	1.00E+00	0.98	0.95	0.89	6.13E-01	3.92E-01	3.21E-02
Xanthosine	1.15	1.19	1.19	9.03E-03	6.70E-03	6.70E-03	1.08	1.11	1.10	6.25E-03	9.58E-04	5.15E-03
Glutathione	1.13	1.09	1.20	2.66E-01	5.16E-01	2.56E-01	1.17	1.05	0.97	7.14E-01	7.14E-01	7.36E-01
CMP	1.12	1.09	1.10	3.34E-02	5.92E-02	9.52E-02	1.01	0.99	0.98	7.46E-01	7.46E-01	7.46E-01
dAMP	1.08	1.09	1.12	1.37E-01	1.14E-01	1.14E-01	1.13	1.10	1.10	1.16E-02	7.15E-03	7.15E-03
3'-O-methylguanosine	1.35	1.32	1.38	2.42E-02	2.35E-02	2.35E-02	1.09	1.01	1.04	2.87E-03	3.53E-01	7.64E-02
AMP	1.14	1.14	1.13	3.66E-02	3.66E-02	3.66E-02	1.04	1.03	1.02	1.69E-01	2.20E-01	2.20E-01
GMP	1.21	1.20	1.21	1.80E-03	1.90E-03	1.80E-03	1.09	1.05	1.04	3.09E-02	3.43E-01	3.43E-01
CDP-Ethanolamine	1.23	1.23	1.24	1.21E-03	1.39E-02	1.30E-02	1.21	1.18	1.20	9.61E-02	2.47E-02	2.47E-02
CDP-choline	1.39	1.37	1.39	5.33E-05	1.77E-04	2.16E-03	1.21	1.19	1.19	1.30E-02	9.62E-04	9.62E-04
ADP-ribose	1.35	1.35	1.35	1.19E-02	1.19E-02	1.19E-02	1.03	1.02	1.02	5.62E-01	9.54E-01	5.62E-01
Uridine 5'-diphosphate (UDP)	1.10	1.10	1.10	4.84E-02	3.37E-02	6.49E-03	1.07	1.03	1.01	8.63E-01	8.63E-01	9.79E-01
UDP-D-Glucose	1.13	1.09	1.19	7.26E-02	2.12E-01	7.26E-02	1.10	1.06	1.05	2.34E-01	2.34E-01	8.56E-01
UDP-D-glucuronate	1.44	1.36	1.45	1.21E-03	1.39E-02	1.39E-02	1.12	1.14	1.14	2.34E-01	3.00E-02	2.49E-01
UDP-N-acetylglucosamine	1.30	1.26	1.32	6.69E-03	7.28E-02	3.68E-02	1.16	1.15	1.17	5.95E-01	1.46E-01	5.95E-01
L-Fucose-1-phosphate	1.10	1.09	1.30	1.37E-01	2.83E-02	7.08E-02	1.12	1.14	1.02	3.07E-01	4.02E-03	3.16E-01
CMP-N-acetylneuraminate	1.14	1.14	1.13	1.49E-03	1.49E-03	2.13E-02	1.06	1.04	1.04	1.14E-01	9.70E-02	9.70E-02
GDP-glucose	1.23	1.27	1.24	8.53E-02	1.06E-01	6.40E-02	1.14	1.14	1.13	6.91E-01	1.53E-01	6.91E-01
FAD	1.14	1.13	1.13	8.60E-03	3.54E-02	3.54E-02	1.07	1.04	1.03	3.09E-02	3.92E-01	4.03E-01
Xanthine	1.11	1.03	1.09	1.75E-02	1.63E-01	2.45E-02	1.00	0.95	0.93	7.49E-01	7.65E-03	7.65E-03
Xanthosine	1.20	1.26	1.17	8.98E-02	8.97E-02	8.97E-02	1.09	1.14	1.13	3.64E-02	9.30E-03	3.64E-02

	womenLCC						menLCC											
	Fold change			p value			Fold change			p value				Δ				
Metabolite	S I/Normal	S II/Normal	S III/Normal	S I/Normal	S II/Normal	S III/Normal	S I/Normal	S II/Normal	S III/Normal	S I/Normal	S II/Normal	S III/Normal	m/z (Experimental)	m/z	ID comment	UPLC	Ionization mode	MSI level
Glutamate	1.10	1.10	1.09	3.39E-03	1.19E-02	9.26E-04	1.07	1.07	1.07	3.27E-06	1.58E-05	7.33E-05	146.04462	8.51	MS1,RT, MSMS match	HILIC	Negative	1
Glutamine	1.05	1.04	1.04	1.07E-01	3.59E-01	1.07E-01	1.03	1.02	1.02	7.87E-02	7.13E-02	1.82E-01	147.06941	4.34	MS1,RT, MSMS match	HILIC	Negative	1
Aspartate	1.16	1.16	1.16	4.18E-04	1.20E-03	6.24E-06	1.11	1.11	1.11	1.41E-04	1.41E-04	2.06E-04	114.01741	0.98	MS1,RT, MSMS match	HILIC	Negative	1
Asparagine	1.29	1.20	1.23	1.07E-01	1.31E-01	2.21E-02	1.08	1.06	1.08	2.45E-02	1.18E-01	1.18E-01	155.03513	0.55	MS1,RT, one fragment match	HILIC	Negative	1
Dimethylglycine	1.07	1.09	1.06	1.01E-01	7.64E-04	9.44E-03	1.10	1.09	1.11	2.21E-03	2.21E-03	3.46E-03	102.05593	1	MS1,RT match, frag<50 Da	HILIC	Negative	1
Serine	1.12	1.09	1.10	5.07E-02	5.34E-02	2.21E-02	1.07	1.07	1.06	6.82E-03	1.70E-02	9.62E-03	104.03142	8.1	MS1,RT, MSMS match	HILIC	Negative	1
Uracil	1.07	1.07	1.07	1.18E-01	2.40E-02	2.40E-02	1.06	1.06	1.07	5.35E-06	4.00E-05	4.72E-06	111.01821	3.52	MS1,RT match, frag<50 Da	HILIC	Positive	1
Creatinine	0.89	0.94	0.91	4.03E-03	4.03E-03	7.37E-04	0.89	0.90	0.92	7.79E-04	4.85E-04	1.79E-03	114.06554	5.8	MS1,RT match	HILIC	Positive	1
succinate	1.02	1.03	1.03	8.21E-01	6.64E-01	6.64E-01	1.02	1.04	1.02	2.36E-01	1.71E-02	4.04E-01	117.019	2.5	MS1,RT, MSMS match	HILIC	Negative	1
Threonine	1.16	1.14	1.14	1.18E-01	1.18E-01	3.47E-02	1.06	1.02	1.06	1.03E-01	3.99E-01	1.03E-01	118.04927	0.82	MS1,RT, MSMS match	HILIC	Negative	1
Hypoxanthine	1.05	1.04	1.04	6.00E-04	4.63E-04	4.63E-04	1.03	1.02	1.02	9.89E-05	8.34E-03	1.28E-04	119.03564	3.7	MS1,RT, MSMS match	HILIC	Negative	1
Taurine	1.08	1.09	1.08	6.19E-06	1.16E-07	1.16E-07	1.08	1.07	1.07	6.18E-08	2.45E-07	6.27E-05	124.00592	10.21	MS1,RT, MSMS match	HILIC	Negative	1
Ornithine	1.08	1.06	1.07	8.99E-02	1.60E-01	8.99E-02	1.03	1.03	1.03	1.28E-01	1.28E-01	1.28E-01	131.08099	3.97	MS1,RT, MSMS match	HILIC	Negative	1
Histidine	1.17	1.12	1.13	1.77E-01	1.95E-01	9.02E-02	1.05	1.05	1.09	2.25E-02	1.23E-01	2.25E-02	156.07019	2.01	MS1,RT, MSMS match	HILIC	Negative	1
Citrulline	1.06	1.07	1.12	3.45E-01	3.45E-01	3.45E-01	1.07	1.05	1.08	2.80E-01	3.09E-01	9.63E-02	174.08358	1.29	MS1,RT, MSMS match	HILIC	Negative	1
Lysine	1.08	1.06	1.07	1.38E-01	1.72E-01	1.38E-01	1.03	1.03	1.03	2.64E-01	5.17E-01	2.64E-01	145.0985	1.5	MS1,RT, MSMS match	HILIC	Negative	1
Alanine	1.16	1.19	1.13	1.59E-02	5.34E-02	3.48E-02	1.18	1.14	1.20	2.77E-04	2.43E-04	2.43E-04	88.03832	6.34	MS1,RT, MSMS match	HILIC	Negative	1
Methionine	1.08	1.05	1.07	5.07E-02	1.60E-01	5.07E-02	1.06	1.04	1.04	2.03E-02	4.91E-02	4.91E-02	148.04323	3.5	MS1,RT, MSMS match	HILIC	Negative	1
Adenine	1.10	1.08	1.07	3.38E-02	9.41E-03	9.41E-03	1.06	1.05	1.03	1.74E-01	6.75E-02	5.38E-01	136.06105	5.5	MS1,RT, MSMS match	HILIC	Negative	1
N-Formylmethionine	1.18	1.14	1.09	2.78E-04	2.24E-05	4.64E-02	1.07	1.09	1.07	4.66E-04	1.27E-06	4.66E-04	176.03925	3.3	MS1,RT, one fragment match	HILIC	Negative	1
Acetyl-lysine	1.43	1.33	1.41	8.51E-02	8.51E-02	8.20E-03	1.29	1.22	1.25	2.93E-02	8.27E-02	3.46E-02	187.10792	4.6	MS1,RT, MSMS match	HILIC	Negative	1
NG,NG-dimethyl-L-arginine (ADMA)	1.14	1.12	1.14	1.01E-01	9.03E-02	2.57E-02	1.08	1.07	1.07	1.32E-03	1.00E-02	1.32E-03	203.15015	0.65	MS1,RT, MSMS match	HILIC	Positive	1
Argininosuccinic acid	1.30	1.28	1.24	6.17E-02	7.61E-02	6.17E-02	1.11	1.11	1.15	5.51E-03	4.91E-02	1.17E-02	291.12204	0.54	MS1,RT, MSMS match	HILIC	Negative	1
Glucose 6-phosphate	1.15	1.12	1.14	3.39E-03	1.85E-02	3.25E-03	1.23	1.12	1.14	1.93E-07	9.19E-07	2.05E-07	259.02031	1.1	MS1,RT, MSMS match	HILIC	Negative	1
Fructose 6-phosphate	1.25	1.25	1.26	2.26E-03	2.32E-04	2.32E-04	1.29	1.25	1.27	4.21E-07	2.88E-06	5.35E-05	241.00322	2.17	MS1,RT, MSMS match	HILIC	Negative	1
Phosphoenolpyruvate	1.09	1.06	1.04	4.00E-01	1.25E-01	4.00E-01	1.06	1.05	1.00	3.12E-01	8.84E-02	4.97E-01	148.96421	1.8	MS1,RT, MSMS match	HILIC	Negative	1
DHAP/G3P	1.07	1.08	1.06	2.69E-02	5.34E-02	2.69E-02	1.09	1.08	1.06	2.03E-02	2.55E-02	1.04E-01	229.01096	11.26	MS1,RT, MSMS match	HILIC	Negative	1
Lactate	1.01	1.00	1.00	5.70E-01	5.70E-01	5.70E-01	1.06	1.05	1.04	2.80E-04	2.80E-04	2.50E-03	89.02333	12	MS1,RT, MSMS match	HILIC	Negative	1
Ribulose 5-phosphate	1.20	1.21	1.20	8.86E-03	1.19E-03	9.26E-04	1.07	1.03	1.03	3.50E-02	2.74E-01	1.88E-01	**211.0006**	**3.15**	MS1,RT, MSMS match	HILIC	Negative	1
Glucosamine 6-phosphate	1.11	1.15	1.11	8.51E-02	8.51E-02	8.51E-02	1.13	1.08	1.07	2.45E-02	1.03E-01	2.56E-01	258.02827	4.77	MS1, MSMS match	HILIC	Negative	1
S-Adenosylhomocysteine	1.25	1.24	1.21	1.80E-03	3.24E-03	3.86E-03	1.18	1.12	1.05	7.65E-03	4.85E-04	2.82E-01	385.1193	1.78	MS1,RT, MSMS match	HILIC	Negative	1
S-Adenosylmethionine	1.43	1.48	1.38	1.13E-02	1.79E-04	2.71E-05	1.25	1.18	1.27	3.21E-02	2.83E-02	3.21E-02	399.13785	2	MS1,RT, MSMS match	HILIC	Negative	1
Spermidine	1.10	1.06	1.07	1.78E-02	1.78E-02	4.36E-02	1.03	1.04	1.08	9.98E-02	9.98E-02	2.05E-03	146.1643	6	MS1, MSMS match	RPLC	Positive	2
Acetylspermine	1.22	1.21	1.19	8.86E-03	9.71E-04	5.78E-03	1.00	1.04	1.13	1.40E-01	1.13E-01	5.37E-04	245.234	1.6	MS1, MSMS match	RPLC	Positive	2
Diacetylspermine	1.13	1.23	1.17	5.99E-02	4.40E-03	6.83E-03	1.00	1.02	1.12	7.33E-01	7.33E-01	1.78E-01	269.23047	6	MS1, MSMS match	RPLC	Positive	2
Glycerol 3-phosphate	0.96	0.95	0.96	1.58E-01	1.58E-01	1.58E-01	0.93	0.94	0.96	2.89E-02	5.76E-03	2.89E-02	**155.0099**	**3.4**	MS1, MSMS match	RPLC	Positive	2
Palmitic acid	1.09	1.09	1.11	3.04E-01	9.03E-02	6.96E-02	1.00	0.99	0.97	8.99E-01	6.10E-01	4.85E-01	239.23634	2.5	MS1, MSMS match	RPLC	Positive	2
Oleic acid	1.04	1.03	1.02	8.37E-01	8.37E-01	8.37E-01	0.94	0.97	0.96	1.14E-02	2.25E-02	1.56E-02	265.2528	0.7	MS1, MSMS match	RPLC	Positive	2
Stearic acid	1.02	1.06	1.06	6.46E-01	1.04E-01	1.04E-01	1.07	1.10	1.08	5.80E-03	5.80E-03	5.80E-03	283.26496	2.6	MS1, MSMS match	RPLC	Positive	2
Carnitine	1.02	1.02	1.01	3.38E-02	3.91E-03	7.37E-04	1.01	1.01	1.01	6.59E-03	6.59E-03	8.86E-02	162.11322	4.5	MS1,RT, MSMS match	HILIC	Positive	1
Palmitoylcarnitine	1.07	1.07	1.06	1.13E-02	9.40E-03	1.13E-02	1.02	1.01	1.02	3.49E-01	6.74E-01	6.74E-01	400.342	0.3	MS1, MSMS match	RPLC	Positive	2
Stearoylcarnitine	1.46	1.42	1.43	2.45E-02	2.45E-02	4.77E-03	1.07	1.02	1.05	7.66E-01	7.66E-01	7.66E-01	450.3554	0.2	MS1, MSMS match	RPLC	Positive	2
LysoPE(16:1)	1.06	1.06	1.06	1.79E-02	1.10E-02	1.10E-02	1.09	1.04	1.05	2.80E-01	3.42E-01	2.06E-01	452.2765	1.5	MS1, MSMS match	RPLC	Positive	2
LysoPE(16:0)	0.98	0.98	0.99	8.53E-01	8.53E-01	8.53E-01	0.93	0.95	0.99	1.17E-01	7.94E-02	3.23E-01	476.2738	2.2	MS1, MSMS match	RPLC	Positive	2
LysoPE(18:2)	1.02	1.04	1.02	4.86E-01	4.81E-01	4.86E-01	0.98	0.98	1.01	4.62E-01	4.62E-01	9.12E-01	478.2938	**2**	MS1, MSMS match	RPLC	Positive	2
LysoPE(18:1)	1.00	1.03	1.00	6.84E-01	6.84E-01	6.84E-01	1.00	0.98	0.99	6.93E-01	6.93E-01	6.93E-01	480.3102	3.3	MS1, MSMS match	RPLC	Positive	2
LysoPC(16:1)	1.05	1.05	1.06	2.80E-01	1.59E-01	1.59E-01	1.03	1.01	1.02	6.60E-01	8.47E-01	6.60E-01	494.3238	0.7	MS1, MSMS match	RPLC	Positive	2
LysoPC(18:1)	1.05	1.05	1.05	5.66E-01	5.15E-01	5.15E-01	0.97	0.99	1.00	7.76E-01	7.76E-01	7.76E-01	504.3442	1.2	MS1, MSMS match	RPLC	Positive	2
LysoPE(18:0)	0.98	1.02	1.02	9.74E-01	8.05E-01	8.05E-01	0.98	0.99	1.03	3.61E-01	3.61E-01	8.81E-01	504.3075	2.6	MS1, MSMS match	RPLC	Positive	2
LysoPE(20:1)	1.01	1.01	0.99	1.00E+00	1.00E+00	1.00E+00	0.98	0.97	1.01	9.14E-01	8.68E-01	9.24E-01	506.3257	**1.1**	MS1, MSMS match	RPLC	Negative	2
LysoPE(22:5)	0.93	0.89	0.93	2.15E-01	5.66E-01	2.15E-01	0.89	0.91	0.95	1.34E-02	1.34E-02	1.82E-01	548.2712	**8.5**	MS1, MSMS match	RPLC	Negative	2
PE(34:1)	1.07	1.03	1.02	1.20E-01	2.28E-01	3.20E-01	1.09	1.08	1.13	1.17E-02	7.87E-03	7.87E-03	716.52618	3.7	MS1, MSMS match	RPLC	Negative	2
PE(36:1)	1.02	1.00	1.00	8.05E-01	8.93E-01	9.50E-01	1.05	1.06	1.09	2.04E-03	1.48E-03	6.19E-04	726.5461	2.5	MS1, MSMS match	RPLC	Negative	2
O-Phosphoethanolamine	1.18	1.16	1.13	2.26E-03	2.26E-03	3.59E-04	1.09	1.10	1.09	3.79E-03	1.58E-03	3.48E-02	164.00193	2.79	MS1,RT match, frag<50 Da	HILIC	Positive	1
Stearoylethanolamide	0.99	1.00	1.01	8.79E-01	8.79E-01	8.79E-01	0.95	0.95	1.00	2.08E-01	8.84E-02	5.62E-01	310.3098	**1.8**	MS1, MSMS match	RPLC	Positive	2
Glucuronate	1.03	0.98	0.98	8.83E-01	4.87E-01	4.87E-01	0.90	0.89	0.92	2.58E-01	1.71E-02	2.58E-01	193.035	**1.8**	MS1,RT, MSMS match	HILIC	Negative	1
Sphinganine-1-phosphate	1.00	0.96	0.96	1.00E+00	1.00E+00	1.00E+00	0.93	0.94	1.00	2.09E-01	9.32E-02	4.81E-01	**364.2604**	**2.4**	MS1, MSMS match	RPLC	Positive	2
Pantothenate	1.05	1.05	1.05	6.17E-02	1.45E-01	6.17E-02	1.07	1.06	1.06	1.31E-01	1.31E-01	1.31E-01	218.102	**6.3**	MS1,RT, MSMS match	HILIC	Negative	1
Vitamin E	1.12	1.14	1.12	2.46E-01	4.80E-02	2.46E-01	1.02	0.98	0.94	8.56E-01	4.49E-01	3.32E-01	429.3756	**4.2**	MS1,RT, MSMS match	HILIC	Negative	1
Cytidine	1.02	1.01	1.01	1.00E+00	1.00E+00	1.00E+00	0.90	0.89	0.90	9.13E-03	9.13E-03	2.12E-02	242.07875	2.2	MS1,RT, MSMS match	HILIC	Positive	1
Deoxyinosine	1.18	1.20	1.19	1.01E-01	1.11E-02	3.95E-03	1.10	1.10	1.05	2.38E-01	3.88E-01	5.30E-01	251.07008	0.29	MS1,RT, MSMS match	HILIC	Negative	1
Adenosine	1.02	1.05	1.00	8.69E-01	8.60E-01	8.60E-01	0.95	1.01	0.96	1.00E+00	1.00E+00	1.00E+00	268.10616	7.8	MS1,RT, MSMS match	HILIC	Positive	1
Xanthosine	1.18	1.18	1.14	1.60E-02	1.60E-02	1.60E-02	1.11	1.09	1.07	4.74E-02	4.74E-02	4.74E-02	283.0639	2.18	MS1,RT, MSMS match	HILIC	Negative	1
Glutathione	1.36	1.07	1.21	3.39E-02	2.48E-01	2.48E-01	1.11	1.08	1.04	5.84E-01	5.89E-01	7.76E-01	306.07635	0.5	MS1, MSMS match	HILIC	Positive	1
CMP	1.09	1.07	1.08	4.98E-02	4.98E-02	4.98E-02	1.01	1.01	1.02	8.56E-01	8.56E-01	8.56E-01	322.04213	2.02	MS1,RT, MSMS match	HILIC	Negative	1
dAMP	1.14	1.11	1.10	9.03E-02	9.03E-02	9.57E-02	1.17	1.12	1.11	1.29E-04	1.29E-04	7.08E-03	332.07614	2	MS1,RT, MSMS match	HILIC	Negative	1
3'-O-methylguanosine	1.37	1.37	1.38	1.38E-01	9.03E-02	3.47E-02	1.12	1.04	1.02	4.93E-02	3.10E-01	3.84E-01	336.06308	0.3	MS1,RT, MSMS match	HILIC	Negative	1
AMP	1.17	1.13	1.15	8.86E-03	7.96E-03	1.16E-03	1.04	1.03	1.01	1.18E-01	2.56E-01	2.71E-01	346.05342	2.63	MS1,RT, MSMS match	HILIC	Negative	1
GMP	1.25	1.20	1.22	3.04E-03	1.80E-03	9.39E-05	1.10	1.07	1.08	1.76E-03	1.44E-01	1.06E-02	362.04711	1.83	MS1,RT, MSMS match	HILIC	Negative	1
CDP-Ethanolamine	1.25	1.25	1.26	3.38E-02	3.69E-04	3.69E-04	1.26	1.20	1.24	7.44E-03	4.06E-04	2.68E-03	445.05125	1.34	MS1, MSMS match	HILIC	Positive	1
CDP-choline	1.35	1.42	1.40	1.43E-02	1.55E-05	3.50E-05	1.22	1.19	1.23	3.10E-03	5.95E-05	2.68E-04	487.09569	2.41	MS1,RT, MSMS match	HILIC	Positive	1
ADP-ribose	1.40	1.32	1.34	7.94E-03	4.72E-02	7.94E-03	1.03	1.01	1.03	1.39E-01	8.60E-01	1.93E-01	542.06879	0.76	MS1,RT, MSMS match	HILIC	Positive	1
Uridine 5'-diphosphate (UDP)	1.11	1.18	1.13	6.77E-03	1.85E-02	1.10E-02	1.16	1.06	1.09	6.13E-03	1.55E-01	3.48E-02	**384.9866**	**5.9**	MS1,RT, MSMS match	HILIC	Negative	1
UDP-D-Glucose	1.20	1.19	1.12	1.77E-01	3.56E-02	4.31E-01	1.09	1.02	1.07	6.34E-01	7.99E-01	6.34E-01	565.04093	1.54	MS1,RT, MSMS match	HILIC	Positive	1
UDP-D-glucuronate	1.49	1.47	1.50	3.38E-02	1.47E-03	1.47E-03	1.20	1.14	1.20	1.64E-02	1.64E-02	1.64E-02	579.02402	3.35	MS1,RT, MSMS match	HILIC	Positive	1
UDP-N-acetylglucosamine	1.38	1.43	1.42	1.61E-01	1.29E-02	1.29E-02	1.27	1.15	1.18	3.21E-02	2.96E-03	3.21E-02	606.06976	0.5	MS1,RT, MSMS match	HILIC	Negative	1
L-Fucose-1-phosphate	1.28	1.20	1.24	1.30E-04	9.58E-04	2.73E-03	1.20	1.14	1.11	1.98E-03	1.71E-03	3.85E-03	243.0285	**4.2**	MS1,RT, MSMS match	HILIC	Negative	1
CMP-N-acetylneuraminate	1.15	1.15	1.17	1.80E-03	3.86E-03	3.86E-03	1.06	1.04	1.04	1.34E-02	2.95E-02	4.25E-03	613.13837	2.76	MS1,RT, MSMS match	HILIC	Negative	1
GDP-glucose	1.25	1.34	1.32	2.75E-02	9.41E-03	9.41E-03	1.19	1.15	1.18	3.43E-02	5.36E-03	1.04E-01	621.089	**12**	MS1,RT, MSMS match	HILIC	Negative	1
FAD	1.14	1.14	1.11	2.23E-02	1.53E-02	1.53E-02	1.04	1.05	1.07	9.89E-02	9.89E-02	9.89E-02	786.15233	3.87	MS1,RT, MSMS match	HILIC	Negative	1
Xanthine	1.09	1.06	1.06	3.31E-01	3.31E-01	3.31E-01	0.96	0.92	0.94	1.75E-02	2.35E-03	3.76E-02	135.03	8.7	MS1, MSMS match	RPLC	Positive	2
Xanthosine	1.18	1.14	1.17	1.28E-01	5.79E-01	1.16E-01	1.14	1.07	1.07	3.49E-01	3.90E-01	3.90E-01	283.0639	2.18	MS1,RT, MSMS match	HILIC	Negative	1

Fold change: median value in tumor tissue divided by median value in normal tissue.

p value: nonparametric Kruskal–Wallis rank sum test with pairwise Wilcoxon Mann-Whitney U test, p values adjusted for false discovery rates (FDR) (Benjamini-Hochberg).
